# A theoretical description of the tree cricket ear as a highly phase sensitive biomechanical interferometer

**DOI:** 10.1016/j.isci.2026.117431

**Published:** 2026-09-04

**Authors:** Emine Celiker, Gregory Patrick Sutton, Natasha Mhatre

**Affiliations:** 1 School of Engineering, University of Leicester, University Road, Leicester LE1 7RH, UK; 2 School of Natural Sciences, University of Lincoln, UK, Joseph Banks Laboratories, Green Lane, Lincoln LN6 7DL, UK; 3 Department of Biology, University of Western Ontario, London, ON N6A 5B7, Canada

**Keywords:** biological interferometer, Ensifera, reduced-order model, insect auditory biomechanics

## Abstract

Sound localization is essential for insect survival, enabling them to pinpoint predators and conspecifics. However, their minute size hinders the use of inter-aural time or intensity differences, a strategy employed by mammals for localizing sound. In this study, we numerically demonstrate that tree cricket hearing systems can act as an interferometer to support sound localization by first splitting the sound wave into two parts, sending one wave to an anterior tympanic membrane and sending the second to a posterior tympanic membrane. The membranes then carry the split waves to a common point, the tracheal wall, where the re-assembly of the wave causes mechanical interference that is sensitive to microsecond time delays. This principle, interferometry, is usually thought of in terms of light and can measure extremely small time delays. Tree cricket ears are equipped to use the same principle as the light interferometer, except with sound, to measure extremely precise locations.

## Highlights

• A numerical model of a tree cricket ear shows an interferometer-like mechanism
• Each ear’s mechanical linkages are sensitive to small differences in acoustic phase
• Phase differences between two eardrums may be generated by sound absorption
• Acousto-mechanical interferometry could enable single-sensor sound localization

## Graphical abstract

**Figure:**
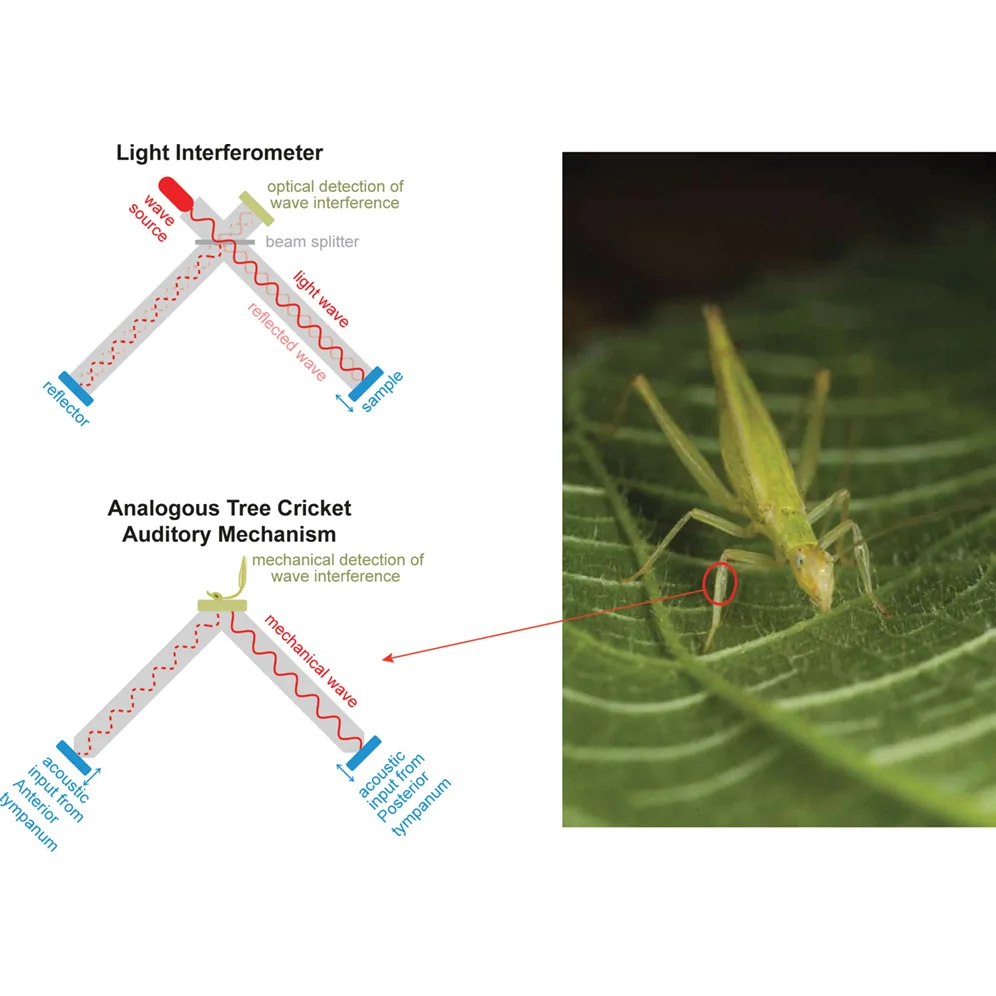

## Teaser

Physiological acoustics; Biological sciences; Zoology; Entomology; Biomechanics

## Introduction

Finding a singing cricket in a living room is infuriatingly difficult. While large vertebrates, like us, can determine the direction of sound from several features that are altered as the sound hits the left and right ears,^1^ resolving this precisely requires the nervous system to react to small (hundreds of microseconds) time differences between the ears.^2^ For crickets, using this mechanism is effectively impossible because their small size would require their nervous system to differentiate between microsecond time differences which are not possible to detect. Yet female crickets have no trouble locating singing males,^3^ even when one ear is removed.^4^^,^^5^ Larger crickets such as field crickets, with a body length of 15–31 mm and a width of 6–8 mm, are thought to solve this problem using a four-input pressure difference system.^6^ However, it is unclear how smaller crickets like tree crickets (body length 11–22 mm and width 1.5–2.5 mm) determine sound direction.

**Figure 1:**
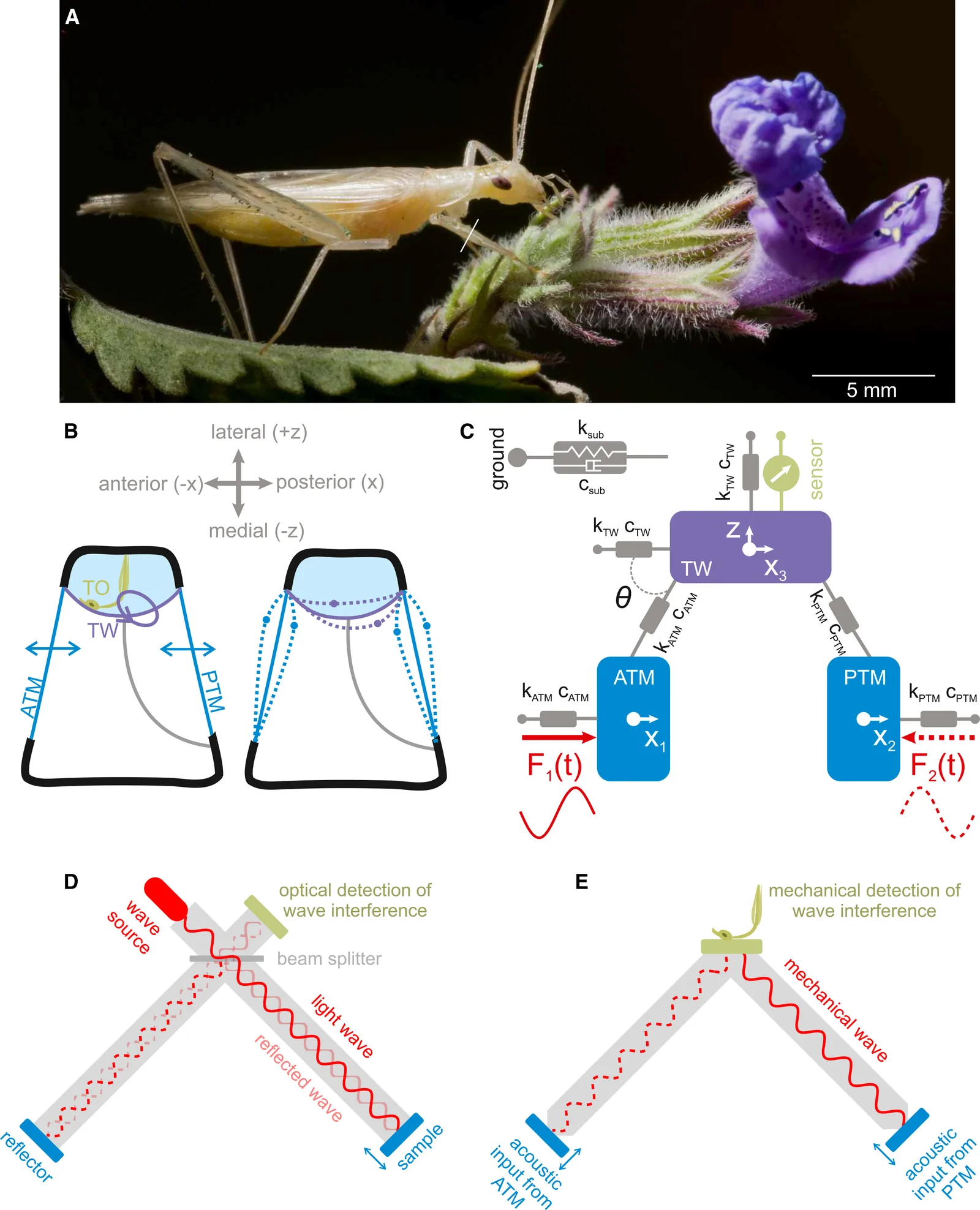
The proposed tree cricket ear biophysical mechanism (A) The tree cricket *O. henryi*. (B) A cross-sectional sketch of the tree cricket *O. henryi* ear near the tympanal end of the acoustic trachea. ATM, anterior tympanic membrane; PTM, posterior tympanic membrane; TO, tympanal organ; TW, trachea wall. The cross-section location indicated by the white line in (A). (C) The fully coupled mechanical system representing the tracheal wall (TW) displacement. The tracheal wall is acted upon by the anterior tympanic membrane, and posterior tympanic membrane. The angle between the tympana and tracheal wall *θ* = 60°.^10^*F*_1_(*t*) and *F*_2_(*t*) represent the harmonic forces of equal magnitude and a frequency of 3 kHz acting on the tympana. (D) A schematic of the light interferometer mechanism. (E) The schematic of the tree cricket ear acting as a biomechanical interferometer.

One of the most well-studied pressure-difference receiver systems is the four-input system of the field crickets.^7^^,^^8^ Tree crickets (Figure 1A), belonging to the same superfamily (Grylloidae) as the field crickets, but a different family (Oecanthidae),^9^ possess ears with a similar anatomical structure. Both the field and tree cricket ears are located in the tibia of their forelegs.^8^^,^^10^ Their tympana are backed by an air-filled tube called the acoustic trachea, with an opening, the acoustic spiracle, located on the thorax. Each Grylloidea ear possesses two tympanic membranes: the anterior tympanic membrane (ATM) and the posterior tympanic membrane (PTM).^8^^,^^10^ In the gryllids, the ATM is highly reduced, whereas in oecanthids, it is equivalent in size to the PTM. The acoustic trachea of each ear is joined by a thin membrane, generating a mechanical connection between the ears.^11^ As a result, their tympana receive multiple inputs of the same sound: directly to the external side, and through the acoustic trachea as well as the thin membrane to the internal side with various pressure amplitudes and phase differences.^7^^,^^8^ While the larger-bodied field crickets (calling song frequency 3–8 kHz) rely on the multiple-input system described earlier for directional hearing, this system is thought to be ineffective for the much smaller tree crickets (calling song frequency 2–7 kHz) due to the wavelength of their calling song frequency being several times larger than their body size.^10^ Hence, the field crickets’ larger bodies allow them to utilize the four-input system effectively, leaving the tree cricket directionality mechanisms unknown.^10^^,^^12^

In addition to differences in calling song frequency and body size, there are also marked differences in the structures of the field cricket and tree cricket tympana. For tree crickets, both ATM and PTM are similar in size, and both move significantly in response to sound.^10^ In this auditory feature, tree crickets are more similar to bush crickets than to field crickets.^13^ Furthermore, both tympana are structurally complex, with ATM containing transverse folds in the central and distal regions, which are much less pronounced and only present at the distal region for the PTM. Consequently, both tympana are corrugated at different levels.^14^ In comparison, the field cricket tympana are not corrugated, and there is a marked difference in the sizes of the ATM and PTM, with PTM being significantly larger than ATM. It was also noted that PTM is the only tympanum that moves significantly in response to sound.^15^ As such, having two tympana per ear does not appear to be a necessary condition for the pressure-difference receiver system to work effectively.

The transduction of the sound vibrations to the mechanosensory neurons is also crucial to sensing. For Grylloidea, the mechanoreceptors are not directly connected to the tympana. Near the tympanal end, the acoustic trachea bifurcates, with the anterior branch containing the ATM and the posterior branch the PTM^16^ (see Figure 1B). Opposed to the two tracheal branches is the auditory vesicle. The auditory vesicle houses the tympanal organ of the insect, which comprises the mechanosensory neurons responsible for transducing sound-driven motion into neural signals. The tympanal organ is directly connected to the internal wall of the auditory vesicle, which is also the external wall of the two tracheal branches. Hence, when the ATM and PTM vibrate, they can transmit the sound vibrations to the trachea wall (TW), leading to its displacement, which in turn activates the sensory receptors.^16^ Consequently, despite not being directly connected to the tympana, mechanoreceptors get activated by a system driven by both the ATM and PTM.^17^

Optical coherence tomography (OCT) is a state-of-the-art non-invasive, high-resolution imaging technique that has recently been applied to record real-time measurements from within hearing systems. OCT measurements of the tree cricket TW displacements have revealed that the transmission of sound vibrations from the tympana to the TW results in the elliptic motion of the wall (Figure 1B).^16^ At first glance, this appears as a mechanical disadvantage as the largest displacement amplitude would occur from a vertical displacement of the TW, leading to the maximal stretching of the neurons, rather than an elliptic trace. Because the TW plays a crucial role in activating sensory receptors, determining the cause of the elliptic motion would explain why the hearing system appears to prefer this movement. Notably, Vavakou et al.’s recent OCT recordings within the bush cricket ear showed a similar elliptic trace of the TW,^18^ and Frost et al.’s OCT recordings within the mammalian ear recorded an elliptic motion of the outer hair cells.^19^ This suggests that the elliptic trace observed in hearing systems is not uncommon and may be functionally adaptive.

We have approximated the mechanical linkages within the tree cricket *Oecanthus henryi* ear between the ATM, PTM, and TW with a three-element mechanical model. The model includes (1) an ATM, which receives one sound input, (2) a PTM, which receives a second sound input, and (3) a TW, which is acted upon through the displacement of each membrane (Figure 1C). This three-element model mechanically recreates the acoustic response of *O. henryi* tracheal wall and the transmission of a mechanical input to the mechanosensory organ. Here, we numerically show that the TW elliptic traces are tied to the phase differences in the sound inputs to the two tympanal membranes. The introduced mechanism has the potential to precisely determine the direction of the sound source. Such phase-dependent interference between the two mechanical inputs detected by the nervous system would make the tree cricket ear a biological example of a mechano-acoustic phase interferometer akin to that developed for light by Michelson and Morley^20^ (Figures 1D and 1E).

Moreover, as this system is sensitive to the phase of the sound (as opposed to the time delay)—the higher the frequency of the sound, the easier it is for the system to mechanically transduce the precise direction of the sound’s source. This would have obvious utility in detecting bats (which have call frequencies in excess of 60 kHz^13^) as well as detecting the song in ultrasonic crickets.^21^

## Results

### ATM response numerical analysis

**Figure 2:**
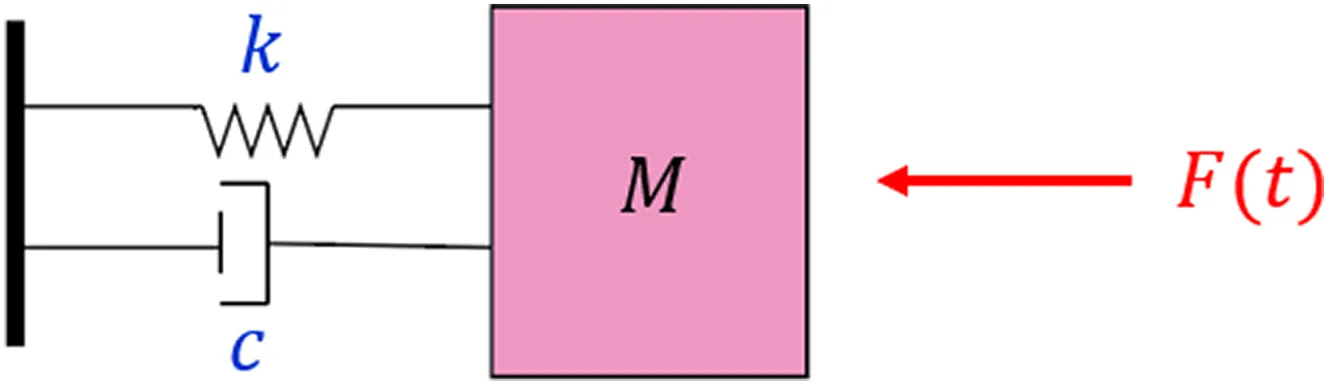
The mass-spring-damper system representing the *O. henryi* tympanum mechanism *M*, *k*, and *c* are the mass, stiffness, and damping parameters of the tympanum, respectively

**Figure 3:**
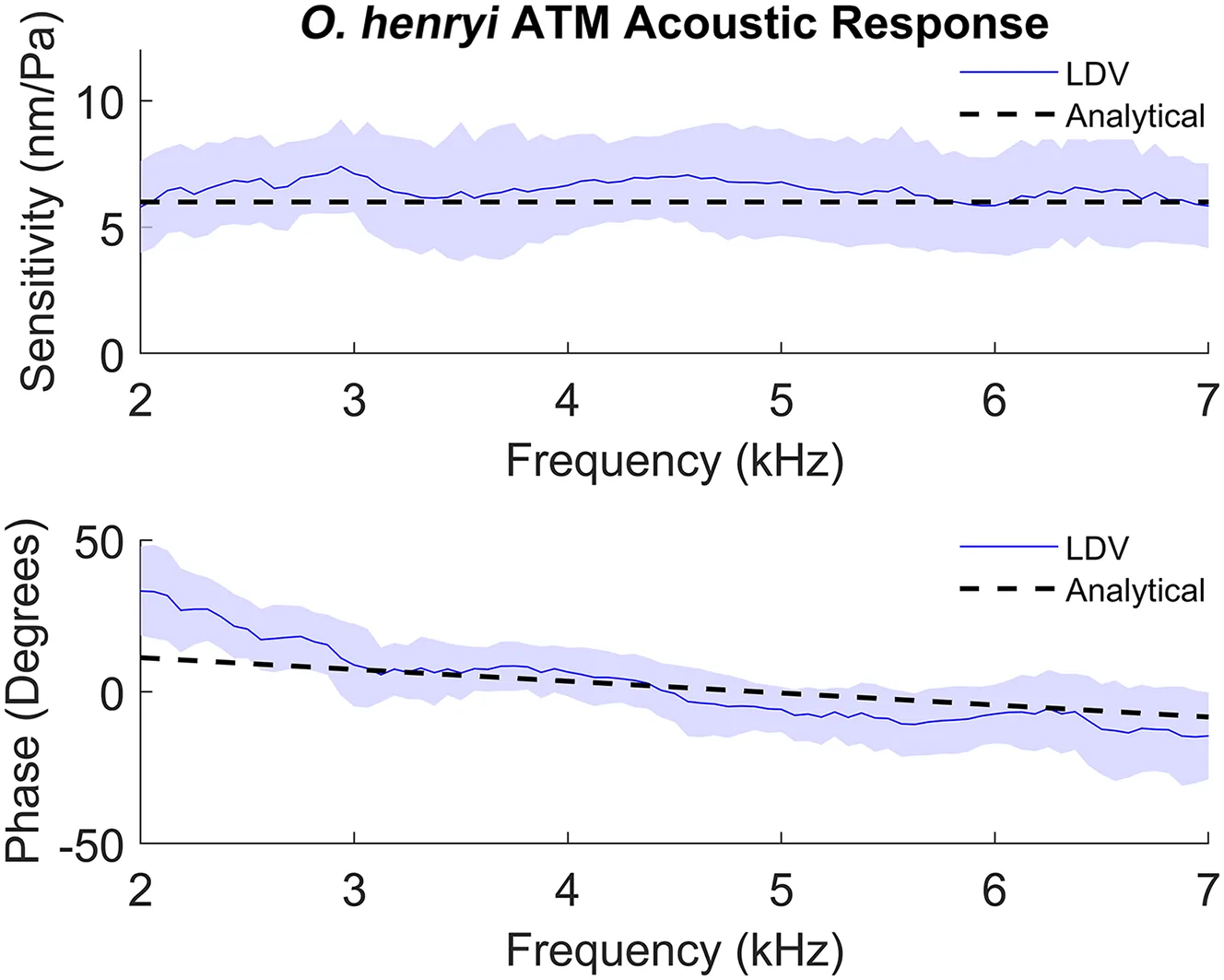
ATM displacement magnitude validation Comparison of the analytical solution and laser Doppler vibrometer (LDV) recordings of tree cricket tympanal displacement magnitude (top) and phase (bottom) from Mhatre and Robert.^12^ Navy blue line shows the mean and shaded zone indicates ±1 SD of the LDV data from *n* = 12 animals.

We first calculated the passive tympanal response to sound pressure in the frequency domain using a lumped element model, within the range 2–7 kHz (see STAR Methods, Figures 1 and 2, and model parameters in Tables 1 and 2). Figure 3 shows a comparison between numerical results and the laser Doppler vibrometer (LDV) recordings.^12^ Consistent with experimental recordings, the solution demonstrated a flat response of the ATM to the sound stimulus across the studied frequency range. The relative percentage error of ATM sensitivity between the mean experimental and numerical data was 11.5% (supplemental information, Methods S1.2). For a direct comparison between the experimental and numerical phase data, the numerical data were calibrated by a magnitude of 20° to account for the fixed time delay in the experimental setup that is not available in the analytical solution. For the frequency range 2.5–7 kHz, the numerical phase calculations remained within the standard deviation bounds of the experimental phase recordings and showed a maximum absolute difference of 28° with the mean LDV recordings (supplemental information, Methods S1.2). Hence, the solution for the ATM sensitivity and phase gave a good match with the LDV recordings.

**Table 1:** ATM dimensions of tree cricket *O. henryi* used to obtain the effective mass, *M*

Parameter	ATM
Length	9 × 10^−4^ m
Width	3.5 × 10^−4^ m
Cross-sectional area (*A*)	2.5 × 10^−7^ m^2^
Thickness (*h*)	2 × 10^−6^ m
Density (*ρ*)	1,200 kg/m^3^
Total Mass (*M*_*tot*_)	5.9 × 10^−10^ kg
Effective Mass (*M*)	1.6 × 10^−10^ kg

**Table 2:** ATM, PTM, and TW material properties used in the mathematical model

Parameter	ATM	PTM	TW
Mass (kg)	1.6 × 10^−10^	1.4 × 10^−10^	2.8 × 10^−10^
Stiffness (N/m)	41.23	41.23	20.62
Damping (kg/s)	0.00047	0.00047	0.00047

### TW elliptic motion numerical analysis

**Figure 4:**
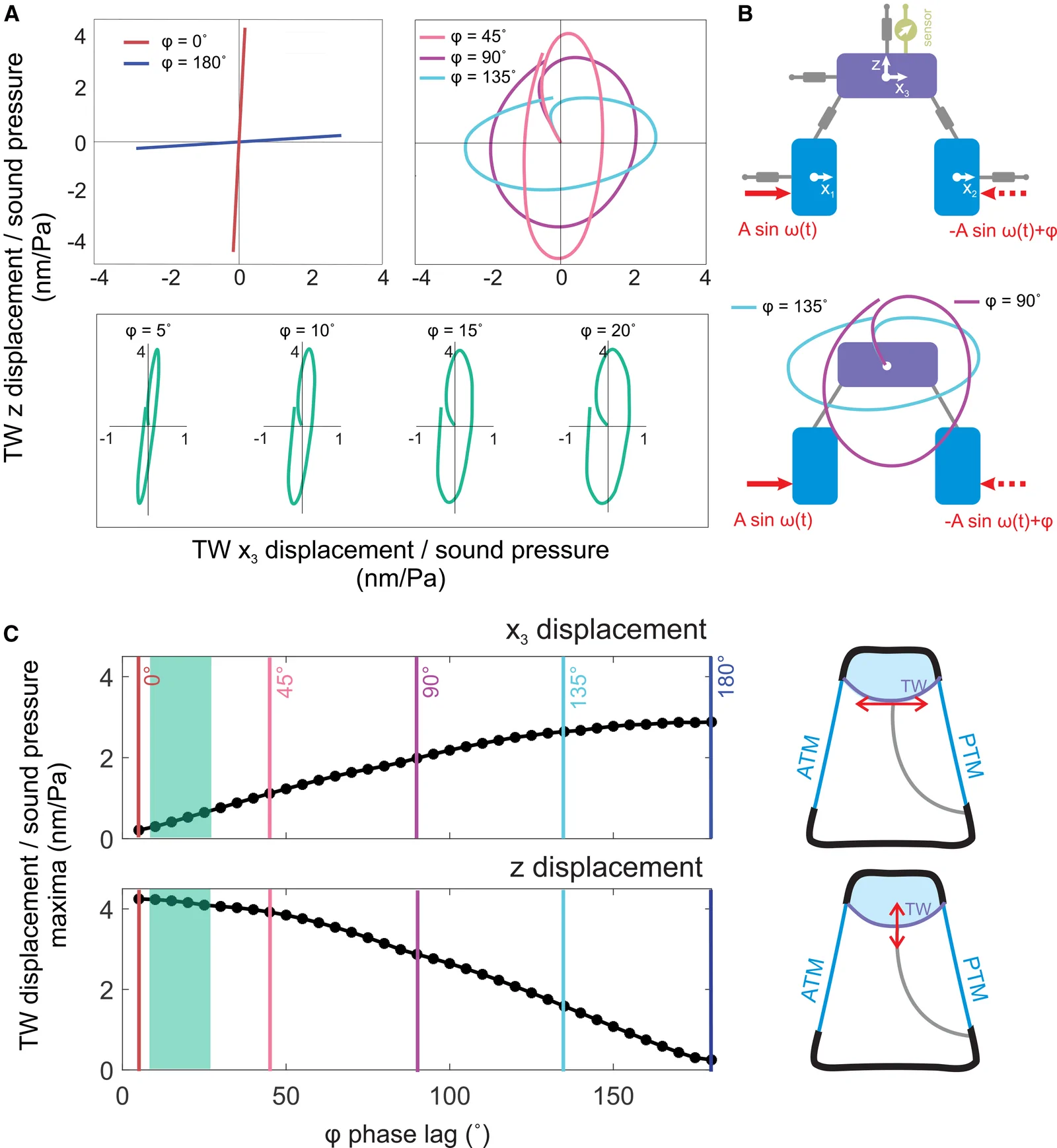
The displacement sensitivity of the tracheal wall at the calling song frequency 3 kHz (at 24°C) of *O. henryi* Phase lag refers to the phase difference between the forces of same magnitude activating the anterior tympanic membrane (ATM), and the posterior tympanic membrane (PTM). (A) The displacement pattern of the tracheal wall when the ATM and PTM are acted on by forces in-phase (phase lag = 0°) and out-of-phase (phase lag = 180°) (top left); The elliptic motion of the tracheal wall for large phase differences between the ATM and PTM (top right); The elliptic motion of the trachea wall for small phase differences between the ATM and PTM (bottom). (B) The lumped element model representing the trachea wall displacement. (C) The displacement magnitude of the tracheal wall in the *x*_3_- and *z*-directions with respect to the phase lag of the forces acting on the ATM and PTM.

To investigate the cause of the observed elliptic motion of the TW in the *xz*-plane (Figure 1B), we utilized the coupled lumped element model described in the STAR Methods section. Simulations were carried out at the *O. henyri* calling song frequency of 3 kHz at 24 °C.^22^ Initially, we modeled the ATM and PTM as being driven by forces of equal magnitude, with either an in-phase (*φ* = 0°) or out-of-phase (*φ* = 180°) relationship. These resulted in a purely vertical (for *φ* = 0°) or purely horizontal (*φ* = 180°) displacement of the TW (Figure 4A [top left]). This outcome did not reproduce the elliptic motion observed in experimental OCT recordings. Subsequently, we explored the effect of unequal force magnitudes, assuming a sound pressure of 0.05 Pa on the ATM and 0.04 Pa on the PTM. However, this also resulted in a similar, non-elliptic displacement pattern (see supplemental information, Figure S4).

Next, we considered the effect of a phase difference between the forces acting on the ATM and PTM on the TW displacement. Following the transmission of vibrations from the tympana which moved with a phase lag of magnitude *φ*, the TW displaced with an elliptic motion in the *xz-*plane (Figure 4). Furthermore, the shape of the ellipse traced by the TW showed a significant dependence on the magnitude of *φ*.

Although recorded using a different tree cricket species (*O. californicus*), a comparison between the OCT measurements of TW displacement magnitude given in the study by Mhatre et al.^16^ for the passive state, and the numerical results (Figure 4) showed a notable match, validating the accuracy of the lumped element model. The ellipse was traced through a clockwise movement of the wall. The change of the phase angle through *φ* = 45°, 90°, 135° showed a difference in the shape of the elliptic motion from a prolate elliptical profile to an oblate elliptical profile (Figure 4A [top right]). With the increase of the phase lag between the ATM and PTM, the magnitude of the horizontal displacement of the TW increased while the vertical displacement decreased (Figure 4C). When a smaller difference of the phase angle was considered (*φ* = 5°, 10°, 15°, 20°), a similar change in the ellipse shape was still present (Figure 4A [bottom]). The numerical results show a widening and shortening of the ellipses by 0.1 nm/Pa per 5-degree phase delay, indicating that the system requires at least a 5-degree phase delay to produce an elliptic motion detectable by the tree cricket. We show an animation of these elliptical motions as a function of phase in supplemental information, Data S1.

A further investigation of TW displacement for *φ* = 45°, 90°, 135° was also considered for unequal sound pressure magnitudes at the ATM and PTM (supplemental information, Methods S4). The obtained results showed that the phase-dependent elliptic trace at the TW was strongly prevalent when there were minor magnitude differences between the ATM and PTM. With the increase of amplitude differences between the ATM and PTM, the elliptic trace tilted further toward the tympanum with the smaller magnitude. However, for larger magnitude differences, the phase-lag dependence of the trace disappeared, and the resulting displacement patterns were aligned for all considered values of *φ* (supplemental information, Figure S5).

We note that for 180° < *φ* < 360°, the elliptic motion of the TW is traced anti-clockwise. Due to the tree cricket calling song having a sinusoidal waveform and the 2*π*-periodicity of the sine function, the same elliptic traces are obtained for phase differences larger than 360° between the tympana.

## Discussion

The proposed biomechanical phase interferometer requires a small number of simple components: (1) two similarly sized membranes transducing disparate vibrational inputs to a common point moving at similar levels, (2) a system that allows for sound direction to create a phase difference between the soundwaves hitting each membrane, and (3) mechanosensory neurons at that common point (the tracheal wall) that respond preferentially to a single direction of movement. The first of these components is a feature common to many Ensiferan species and its functional significance was hitherto unappreciated. For instance, bush crickets (also Ensiferans), have a very similar ear morphology consisting of two membranes carrying the sound to a common point.^23^ For component two, small phase differences between closely placed tympana may be created due to diffraction by the body of the animal, ear anatomy, and the substrate it stands on. Indeed, a directionally dependent phase difference in the sound hitting the ATM and PTM has been observed in bush crickets.^13^ Such a difference may be accentuated by the geometry of the tracheal branches carrying the sound to each membrane.^24^ We believe that only such a phase difference would give rise to an elliptical motion of the TW. Moreover, similar elliptical movements in the TW have been observed in tree crickets^16^ and also in bush crickets,^18^ strongly suggesting that this phase interferometry method is widespread among Ensiferans. Finally, it is also possible that mechanosensory neurons respond preferentially to certain axes of stimulation.^25^^,^^26^ This simplicity provides a huge number of parameters that can then be further tuned to specialize different Ensiferan ears for different inputs. Membrane geometry,^10^^,^^27^ membrane mechanical properties,^28^ membrane anisotropy,^10^^,^^14^^,^^29^ acoustic trachea geometry,^11^^,^^24^^,^^30^ fluid properties within the auditory vesicle,^24^^,^^31^ or mechanisms for active amplification^12^ could all be modified to specialize an interferometer for specific sound inputs.

### Lumped element models of tree cricket ear biomechanism

#### Tympanum

Tree cricket tympana have complex structures with a small responsive region on their surfaces. Within this region, in the passive state, the membranes oscillate at the fundamental mode.^10^^,^^14^ Consequently, we were able to represent the passive state tympanum as a simple oscillator governed by its mass, stiffness, and damping properties.^6^ The mass of the tympanum was scaled down to account for the maximal deflection occurring in the central region of the responsive part of the membrane.^32^ This resulted in a good match between the experimental and numerical data of displacement magnitude between 2 and 7 kHz (Figure 3). The close match between the results shows that a simple mass-spring-damper system is sufficient to capture the passive state response of the tympanum.

LDV recordings showed the passive state tympanum to have a flat response to sound stimulus between 2 and 7 kHz,^10^^,^^12^ suggesting that the system is stiffness dominated within this frequency range.^33^ Since the precise stiffness of the tympanum is not known, we took advantage of its static displacement and calculated the effective stiffness using the analytical solution to the equation of motion governing the tympana biomechanics (Equation 5). As a validation of the effective stiffness, we compared the obtained value with the static stiffness of a clamped circular plate of similar dimensions to the ATM and an elastic modulus of 2 GPa (see supplemental information, Methods S1.1). This calculation resulted in a static stiffness *k*_*s*_ = 41.38 N/m of the plate, which compared remarkably well with the effective stiffness parameter used in the lumped-element model (*k* = 41.23 N/m).

To account for the negative phase slope observed with the LDV recordings, we defined a frequency-dependent damping parameter (*c*(*ω*)) based on parameter sweeps (supplemental information, Methods S1.2). The numerical phase data remained within the standard deviation range of the LDV recordings for the large majority of the considered frequency range (2.5–7 kHz). Furthermore, the numerical phase data have a negative slope consistent with the experimental data, thus demonstrating a good calibration with the compared LDV data.^12^ The discrepancies are attributable to the minor differences in the experimental and model set-up and the inherent complexities of a biological material which were not accounted for in the lumped-element model due to simplifying assumptions. Since the system is stiffness-dominated, the damping values were low as expected (supplemental information, Methods S1.2).

It is noteworthy that although a frequency-dependent effective damping parameter was adopted to calibrate the phase data with the LDV recordings, the parameter sweeps defining *c*(*ω*) were guided by the analytic solution (Equation 5) rather than an empirical equation of best fit (supplemental information, Methods S1.2). Furthermore, *c*(*ω*) showed a negligibly small decrease with the increase of frequency, demonstrating that the system can be efficiently represented by a linear viscous damper. Due to the displacement of the system being independent of frequency, the largest influence of damping was expected to be on the phase response of the ATM, which was verified by parameter sweeps (supplemental information, Methods S1.2). This further highlights the importance of tympanum material properties beyond its resonance.

#### Trachea wall

Following the validation of the tympanum model via comparisons with experimental data, we extended our mechanical system to represent the movement of the TW, which is flanked by the ATM and the PTM (Figure 1B). In this system, we considered the transmission of the tympanal vibrations to the TW via a fully coupled lumped element model (Figure 1C). In particular, we were interested in capturing the empirically observed elliptic motion of the TW in a different *Oecanthus* species.^16^ The movement of the TW plays a major significance in tree cricket sound processing as the mechanosensors are attached to it. Hence, understanding the cause and the function of the elliptic TW motion becomes crucial for a full comprehension of sound transduction within the tree cricket ear.

The numerical results showed that the elliptic motion was not present when the tympana were in-phase (Figure 4A), although this led to the maximum vertical displacement amplitude of the wall (constructive interference in *z-*direction). Similarly, when the tympana were out-of-phase, the elliptic motion was not present, and due to the destructive interference of the ATM and PTM movement in *z-*direction there was no significant vertical displacement observed at the TW. A similar displacement pattern was observed when the sound pressure acting on the ATM and PTM had different magnitudes (supplemental information, Methods S4).

The elliptic motion occurred when there was a phase difference between the tympana of magnitude 0° < *φ* < 180° (Figure 4A). Furthermore, the results showed a change in the ellipse shape dependent on the value of the phase lag between the displacement of the ATM and PTM. This difference was evident even at the smallest phase lag between the ATM and PTM.

Analogous to the analytical solution, the temporal response of the ATM and PTM at 3 kHz, as obtained from the fully coupled lumped element model incorporating the transient effect, gave a flat response. (see supplemental information, Methods S5). This implies that in the passive state, the TW does not have a “push-back” effect on the tympana. The tympana displacement magnitude obtained via the fully coupled model (supplemental information, Figure S5) also compared well in magnitude with the analytical solution to the simplified model of the tympanum (Figure 3). This further suggests that in the passive state, in the absence of active amplification, vibrations are mainly dissipated within the mechanosensory organ.

Similar to the simple ATM lumped-element model, the fully coupled lumped-element model also considered a single input to the tympanum. This simplification allowed for a breakdown of the system to its simplest form for a primary understanding of the cause behind the elliptic TW motion. While the phase delay between the tympanum was modeled to be a consequence of the phase lag between the forces acting on ATM and PTM, the phase difference leading to the experimentally observed elliptic motion is expected to be inherent to the tree cricket ear. Many material and anatomical conditions of the ear can lead to the required phase difference between the ATM and PTM, ranging from differences of their material properties to the asymmetric tracheal branches behind the membranes. Although not investigated in tree crickets, such a phase difference was recorded from the bush cricket ear at higher frequencies,^13^ which was also noted to be directional.

### TW elliptic motion and directional hearing

Elliptic motion of mechanosensory receptors in the locust ear was first reported by Stephen and Bennet-Clark.^34^ Morphologically different than a tree cricket ear, the locust mechanosensory receptor, the Müller’s organ, is coupled to the tympanum via three thin structures. Stephen and Bennet-Clark noted that differences in the phase and amplitude of the thin structures (styliform body and folded body) led to the movement of Müller’s organ depicted as Lissajous’ figures of different sizes. The authors commented that “The advantage of such an arrangement in the locust ear is unclear, particularly as it would appear at first sight that the two drives will interact to reduce the sharpness of tuning of each other by viscous coupling through Müller’s organ.”

The recent observation of the elliptic displacements of the TW in tree crickets^16^ and bush crickets^18^ has further increased the interest in the function of such a trace. Similar to the locust ear, such a movement of the TW may be considered unpropitious from the point of view that they lead to a smaller magnitude in the vertical displacement of the TW (see Figure 4C). Consequently, if the transduction of mechanoreceptors was dependent solely on the vertical displacement magnitude, they would receive less force toward activation. As suggested by Vavakou et al., the transduction of the mechanosensory cells is likely to be driven by the more complex elliptic motion of the TW and the sensory cells, leading to the tilting and pivoting of the sensory dendrite as well as stretching.^18^^,^^35^ Nevertheless, it is worth noting that an elliptic trace provides significant displacement in both *x-* and *z-*axes, as well as directions in-between these primary axes. Hence, this versatile displacement pattern could provide an all-encompassing activation mode to mechano-receptors.

Based on our numerical results which show the phase dependence of the TW motion (Figure 4), we further conjecture that the TW elliptic motion could contribute to the directional hearing of tree crickets with the mechanoreceptors detecting the changes in the elliptic trace. Or, equivalently, by the TW acting as a biomechanical phase interferometer.

Interferometric optical testing is based on the phenomena of interference, or the superposition of two waves, and is the basis for some of the most sensitive measurement equipment ever made.^20^^,^^36^^,^^37^ The resulting interference fringes give information about the difference in optical path lengths and directions.^38^ For monochromatic waves, in simplest terms, the interference can be described by the two-beam interference equation.^39^ For instance, let *y*_1_(*t*) = *A* sin(*ωt*) and *y*_2_(*t*) = *A* sin(*ωt* + *φ*) be two beams of equal magnitude *A* and angular frequency *ω*, with a phase difference of *φ*. Assume they are moving in the directions of *F*_1_(*t*) and *F*_2_(*t*) as given in Figure 1C. Then we can describe the interference magnitude at time *t* in the *x-* and *z-* directions as:

(Equation 1) $$x\left(t\right)=\left(y_{1}\left(t\right)-y_{2}\left(t\right)\right)\cos\left(\theta \right),$$

(Equation 2) $$z\left(t\right)=\left(y_{1}\left(t\right)+y_{2}\left(t\right)\right)\sin\left(\theta \right).$$

**Figure 5:**
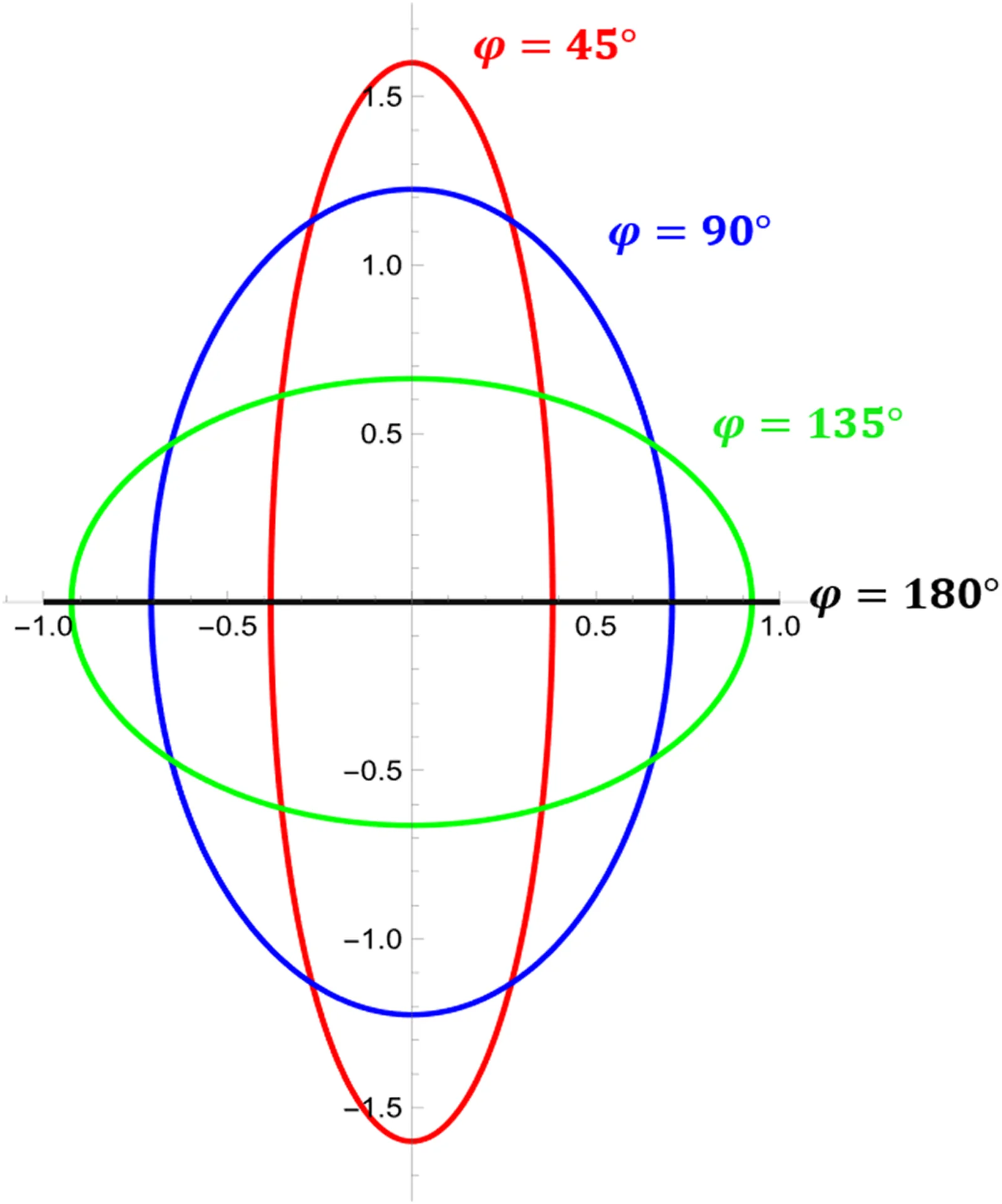
The phase angle dependent elliptic motion obtained in the *xz-*plane from the superposition of two simple waves moving in opposite directions with a phase difference of *φ*.

Letting, for instance, *A* = 1, *ω* = 2*π* × 3000 Hz, and varying the phase angle *φ*, we obtain the parametric plot of the interference in the *xz*-plane as in Figure 5. The qualitative match between the elliptic motion captured at the tracheal wall (Figure 4A) and by using the simplest interference equations (Figure 5) strongly suggests that the tree cricket ear could act as a biomechanical phase interferometer. The elliptic motion of the TW causes the magnitudes of both horizontal and vertical displacements to vary significantly with phase (Figure 4C). Consequently, regardless of the direction by which sensory neurons are activated,^26^ this phase-dependent variation in displacement will lead to a substantial difference in their activation levels. The qualitative robustness of the lumped-element model to parameter variations also suggests that the system is not strictly dependent on an optimal parameter set for directional sensitivity, making it a highly versatile mechanism.

### A biomechanical phase interferometer within the tree cricket and bush cricket hearing systems

As the OCT recordings of TW displacements have shown similar elliptic traces in tree crickets^16^ and bush crickets,^18^ we conjecture that both insect groups have the potential to utilize this unique displacement for sound localization. Similar to tree crickets, bush crickets also possess an auditory vesicle (inner-ear) flanked by the ATM and PTM.^23^ For both species, the ATM and PTM actively take part in transmitting the sound vibrations to the auditory vesicle, although for some bush cricket species this is facilitated by hardened cuticular regions on the tympana (the tympanal plates) acting as impedance converters.^40^ While tree crickets have a four-input system, bush cricket ears do not allow for cross-talk between the trachea, leading to a dual-input system.^41^ Another difference between the insects is that for bush crickets the auditory sensilla are not directly connected to the TW.^42^ However, the mechanoreceptors are still located above the wall, and as demonstrated by advanced finite element analysis the TW acts as the driving force for activating them.^43^ Hence, despite the differences in the anatomy of their ears, the bush cricket ear also possesses the necessary qualities to act as a biomechanical phase interferometer. It is worth noting that, due to the negligible displacement of the field cricket ATM, an interferometric sound localization mechanism is not expected to prevail within their auditory mechanism. As demonstrated by our numerical investigation, the proposed mechanism functions effectively when there is a comparable displacement magnitude from the ATM and PTM of the same ear (supplemental information, Figure S5).

Still, a question that remains is whether the required phase delay between the ATM and PTM exists within the ears of bush and tree crickets for the implementation of the proposed interferometer. Both tree and bush crickets possess ears of minuscule dimensions, with a distance of 100–200 μm between the ATM and PTM of the same ear.^10^^,^^23^ The LDV recordings of external input to the tympana of the bush cricket species *Copiphora gorgonensis* have shown a significant, sound direction-dependent phase delay between the ATM and PTM of the same ear,^13^ which would allow for the proposed interferometer to work efficiently. Furthermore, the internal input to the tympana of the same species has also led to a significant phase delay due to the asymmetry of the tracheal branches delivering the sound wave to the tympana, as obtained from LDV recordings and numerical investigations.^24^ However, the contribution of the asymmetric tracheal branches to directional hearing requires further investigation. Nevertheless, these recordings suggest that the required phase lag between the ATM and PTM exists for bush crickets, although its precise cause remains elusive and warrants further investigation. Despite a lack of similar data for tree crickets, it is plausible that the phase lag also exists within their hearing system.

To test how sound interacting with the body of a tree cricket affects the phase difference between the tree cricket’s ATM and PTM, we numerically simulated it using an idealized geometry of the insect body on a substrate (supplemental information, Methods S7). An absorption coefficient of 0.02 generated a phase difference of 20.07° between the tympana of the ipsilateral ear and 7.93° between the ears, which is of sufficient magnitude for the proposed interferometer to work effectively (supplemental information, Table S4). For an absorption coefficient of 0.2 at high frequencies (>1 kHz), such as can be seen in rigid-framed porous materials,^44^ porous materials,^45^ and even in apparently non-porous natural materials like leaves,^46^ the simulation yielded a phase difference of 117.29° at 3 kHz between the ATM and PTM of the ipsilateral ear (supplemental information, Table S4; Figure S8). This phase difference was reduced to 3.33° at the contralateral ear. These results demonstrate that diffraction taken together with local dissipation of sound generates a sufficient phase difference between the ATM and PTM to utilize the proposed interferometer mechanism. Although it is not known if the chitin in the cuticle is arranged such that it gives porous-like, absorptive acoustic properties, its clear advantage, as observed via the simulations, suggests that this quality merits future investigation.

The FEA results also showed a clear asymmetry in the magnitude of the phase difference between the tympana of the ipsilateral and contralateral ears (supplemental information, Table S4). The asymmetry is especially noteworthy, as it would lead to a difference in the characteristics of the ellipse traced by the TW (Figure 4). Consequently, the displacement magnitude of the TW would differ in all axes, leading to a considerable difference in the displacement of the mechanosensors depending on the arrival of the sound from the ipsilateral or the contralateral. We conjecture that the asymmetric phase difference between the two tympana coupled with an interaural comparison would allow the tree cricket to differentiate between the left and right arrival of the sound with high precision. Whereas an artificial sensor could be designed where the *x* and *z* displacement could be compared intra-aurally to compute the same information.

The FEA model was also used to analyze the incident angle dependence of the phase difference between the ipsilateral tympana within the range 0° ≤ *θ* ≤ 90°, with a resolution of 30°. The phase difference increased with the increase of the incident angle across the insect’s frontal field (0° ≤ *θ* ≤ 60°), with a magnitude large enough to resolve sound direction via the proposed interferometer (supplemental information, Table S5). However, the phase difference decreased for 60° ≤ *θ* ≤ 90°. We attribute this to using an idealized geometry of the insect body within the model, and expect a larger phase difference in reality due to the naturally uneven curvature of the insect’s body.

The FEA simulations were carried out in the frequency range 1.5–7 kHz with a resolution of 1.5 kHz. The results showed a reverse correlation between the increase of frequency and the phase difference between the ipsilateral ATM and PTM (supplemental information, Figure S8). Here, we use a frequency-independent absorption coefficient of magnitude 0.2 to minimize our assumptions. However, in real materials, this coefficient rises with frequency. This would increase the phase variation seen at higher frequencies, reversing the unexpected trend we see.^45^ This inverse relationship further demonstrates how strongly the TW motion can depend on the acoustic properties and geometry of the involved biological materials. Consequently, for a more complete understanding of the proposed mechanism, a more robust FEA model, comprising more accurate acoustic parameters and an experimental validation of the motion becomes essential. Nevertheless, our investigation provides a theoretical framework that strongly supports the existence of the proposed interferometric mechanism within the tree cricket hearing system, opening new grounds for the investigation of directional hearing in these insects.

Given the considerable size difference between the anterior and posterior tracheal branches in tree crickets (Figure 1B), and the observation in bush crickets^24^ that such differences affect sound arrival time, it is reasonable that this disparity contributes to further increase phase differences between the displacements of ATM and PTM. It is also worth noting that while our mathematical model considered only one input to the tympana, the multiple-inputs tree cricket ear receives to the ATM and PTM could further enhance the phase difference between the tympana, thus allowing the proposed biomechanical phase interferometer to function more effectively. Moreover, we assumed that the ATM and PTM both comprised isotropic and homogeneous material. Previous experimental investigations showed that, although more pronounced for ATM, both tympana are structurally complex and have a series of transverse folds on their surfaces.^12^ Based on Equation 6, such a material structure would have the potential to create a phase difference between the tympana which, based on our results, is of high significance for the elliptic motion of the TW. While beyond the scope of this study, the material properties of the tympana are certainly worth further investigation for a more complete understanding of their acoustic properties.

The temperature-dependent *O. henyri* calling song frequency spectrum lies within 2.4–3.3 kHz.^22^ For the passive state response, neither the LDV recordings nor the numerical results showed a natural tuning of the tympanum to this frequency range. Nevertheless, one very unique property of the tree cricket tympanal ear is its ability to perform active amplification and adaptive tuning through physiological nonlinearities at this frequency range.^12^ Through our mathematical model, we only considered the passive state of the tree cricket ear, as observed at high amplitude sound stimulus and postmortem experiments.^10^^,^^16^ Phase changes of the tympanum during active amplification were measured by LDV recordings,^12^ although their influence on sound localization remains elusive. While further investigation is required to quantify the phase difference between the ATM and PTM for different sound directions and tympanal states, our results introduce the possibility of a new mechanism which could allow the tree crickets to localize sound during both passive and active states.

Although the full array of tree cricket auditory mechanoreceptive neurons has not been completely morphologically characterized, FIB-SEM data from Mhatre and Robert^12^ (supplemental information) and Mhatre et al.^16^ suggest that they are oriented similarly to field cricket neurons—a single parallel sheet where all neurons are oriented the same way, but at different depths along the organ. While this suggests that the neurons are not specifically oriented to measure both axes of elliptical motion, this quality is not a necessity for them to interpret the interferometric directionality cues proposed. For the neurons to detect sound location from the TW wall motion, it would suffice if they were more sensitive to a single axis of motion, as has been suggested for insect mechanosensory neurons.^26^

We have trouble locating that singing cricket because of its tonal nature, and our nervous system has difficulty resolving the minute time delays in such signals. Tree crickets, on the other hand, could pinpoint the sound using mechanoacoustic phase interferometry, letting the tympanal membranes and the tracheal wall precisely tell them where a singing conspecific is.

### Limitations of the study

It is worth noting that the effective stiffness of the ATM lumped-element model is “tuned” to represent all parts of the hearing system influencing the movement of the ATM, namely, the impact of the acoustic trachea impedance and the multiple inputs that the ATM receives. Hence, the ATM oscillating at the fundamental mode within its responsive region^10^ allowed us to represent it as a simple oscillator,^6^ albeit with a tuned stiffness.

Furthermore, since the physical parameters in our TW simulations are effective values derived from simplifying assumptions, the resulting conclusions are primarily qualitative. However, the extensive parameter sensitivity analyses we have carried out both on the isolated tympanum (supplemental information, Methods S1) and the fully coupled system (supplemental information, Methods S3 and S6) demonstrate the system’s inherent robustness. The parameter analyses strongly suggest that the passive-state tree cricket ear is governed by a stiffness-dominated mechanism, which retains its qualitative properties within (and likely beyond) a ±25% parameter variation. Hence, the developed reduced-order model provides an informative biophysical mechanism for future, more refined investigations of a mechanism with strong sound localization potential at the micro-scale.

## Resource availability

### Lead contact

Requests for further information and resources should be directed to and will be fulfilled by the lead contact, Emine Celiker (ec403@leicester.ac.uk).

### Materials availability

This study did not generate new materials.

### Data and code availability

• No standardized dataset has been reported in this study.
• All original code has been deposited at Figshare with the https://doi.org/10.6084/m9.figshare.30510512.
• Any additional information required to reanalyze the data reported in this paper is available from the lead contact upon request.

## Acknowledgments

N.M. was supported by a Canada Foundation for Innovation grant (CFI4748), the Ontario Research Fund, NSERC Discovery Grant (687216), early career supplement (675248), and NSERC Canada Research Chair (693206), and the Western SEED grant. G.P.S. was supported by a University Research Fellowship from the Royal Society, UK (UF130507) and by the UK MRC (MR/T046619/1) as part of the NSF/CIHR/DFG/FRQ/UKRI-MRC Next Generation Networks for Neuroscience Program.

## Author contributions

Conceptualization, E.C., G.P.S., and N.M.; methodology, E.C., G.P.S., and N.M.; investigation, E.C., G.P.S., and N.M.; writing – original draft, E.C.; writing – review and editing, E.C., G.P.S., and N.M.; funding acquisition, G.P.S. and N.M.; resources, E.C. and N.M.

## Declaration of interests

The authors declare no competing interests.

## STAR★Methods

### Key resources table

**Table:** 

REAGENT or RESOURCE	SOURCE	IDENTIFIER
**Software and algorithms**		

Wolfram Mathematica 13.3	N/A	https://doi.org/10.6084/m9.figshare.30510512

### Method details

#### Mechanism of tree cricket tympanum

To obtain a qualitative understanding of the mechanical properties of the tree cricket *O. henryi* tympana, we modeled the ATM using a lumped element approach (Figure 2). The model is characterised by an effective mass (*M*), effective stiffness (*k*), and effective damping (*c*).

The tympanal displacement magnitude was obtained from the solution to the equation of motion in the frequency domain,

(Equation 3) $$\left(-M\omega^{2}+jcw+k\right)X\left(\omega \right)=F\left(\omega \right),$$

which is the mathematical representation of the system given in Figure 2. The dependent variable *X*(*ω*) is the complex displacement with respect to the angular frequency (*w* = 2*πf*, *f* = frequency), $j=\sqrt{-1}$, and the externally applied force *F*(*ω*) = *F*_0_ represents the sound stimulus. The magnitude of the applied mechanical force was taken as *F*_0_ = *P*_0_ × *A*, where *P*_0_ = 0.04 Pa and *A* is the cross-sectional area of the membrane (Table 1), which is equivalent to an acoustic pressure of magnitude *P*_0_ = 0.04 Pa acting on the tympanic membrane.^6^ The solution was considered in the frequency range 2–7 kHz.

The analytical solution to Equation 3 takes the well-known form (Ch. 1, pp.10^47^).

(Equation 4) $$X\left(\omega \right)=\frac{P_{0}\times A}{j\omega \left(c+j\left(\omega M-k/\omega \right)\right)},$$

which gives the displacement magnitude as

(Equation 5) $$|X\left(\omega \right)|=\frac{P_{0}\times A}{\omega \sqrt{c^{2}+{\left(\omega M-k/\omega \right)}^{2}}},$$

with the corresponding phase angle

(Equation 6) $$\Theta =\tan^{-1}\left(\frac{-c}{k/\omega -\omega M}\right).$$

Next, the parameter values of *M*, *k* and *c* that fit the known *O. henryi* ATM properties were considered. The total mass of the tympanum was calculated using the formula.

(Equation 7) $$M_{\mathrm{tot}}=A\times h\times \rho ,$$

where *A* = cross-sectional area, *h* = thickness and *ρ* = density of ATM, whose values are listed in Table 1. The ATM was assumed to have an elliptic cross-sectional area, and its dimensions were taken as measured in^10^ and^14^ for *O. henryi*. The density was taken as the mean density value of insect cuticle due to the narrow variation between measurements (cf.^28^).

A laser Doppler vibrometer (LDV) is a non-contact instrument that measures surface vibration velocity and displacement using the Doppler shift of a laser beam. The LDV measurements of the *O. henryi* ATM by Mhatre et al. (2009) demonstrated that only small portions of the membrane are set in motion to sound, with maximal deflection occurring in the central region of the membrane (the fundamental mode).^10^ Making the simplifying assumption that the active region is a uniform clamped circular membrane vibrating at the fundamental mode, we accordingly adopted an effective mass as a fraction of its total mass, namely *M* = 0.27*M*_*tot*_.^32^

For the value of the stiffness parameter *k* of the ATM, we made the simplifying assumption that the tympanum is isoptropic and homogeneous. The ATM displacement with respect to the sound stimulus is independent of frequency in the range 2–7 kHz.^10^^,^^12^ This suggests that the dominant parameter of the system is *k*. Based on this, we make a further simplifying assumption that the system is stiffness dominated in this frequency range (Ch.1, pp.18^47^) to obtain the effective stiffness from the analytical solution Equation 5.

For a stiffness dominated system, the dominant term in the denominator’s square root of Equation 5 becomes *k*/*ω*. As seen from the LDV recordings presented in^10^ and,^12^ in the frequency range 2–7 kHz the sensitivity of the ATM is ∼6 nm/Pa. Using this, the effective stiffness *k* is obtained from the relation

(Equation 8) $$6\times 10^{-9}=\frac{\left|X\left(\omega \right)\right|}{P_{0}}∼\frac{A}{k}\;\text{m/Pa}.$$

Rearranging, we obtain the effective stiffness as

$$k∼\frac{2.474\times 10^{-7}}{6\times 10^{-9}}=41.23\;\text{N/m}.$$

The calculated effective stiffness of *k* = 41.23 N/m for the lumped element model is consistent with the expected membrane stiffness arising from the tympanum’s material properties and physiological pre-tension (see supplemental information, Methods S1.1). Furthermore, as expected from a stiffness dominated system, *k*/*ω* is significantly larger than *ωM*.

While the magnitude of the response is stiffness-dominated, the observed non-zero phase response indicates that damping is non-negligible. To ensure a quantitative match with the LDV recordings, we derived a frequency dependent effective damping parameter, *c*(*ω*) using parameter sweeps based on Equation 5 (see supplemental information, Methods S1.2). By comparing these results with the LDV data^12^ we were able to calibrate the effective parameters to more accurately represent the underlying mechanics of the system. It is worth noting that the calculated *c*(*ω*) showed a very minor dependence on frequency within the considered range (supplemental information, Figure S2), strongly suggesting that the system exhibits behavior consistent with a linear viscous damper.

#### A fully-coupled lumped element model of trachea wall displacement

The OCT recordings of the tree cricket ear by Mhatre et al.^16^ showed that the displacement of the trachea wall (TW) was primarily guided by the tympanic membranes through their mechanical coupling (see Figure 1B). Based on this, we developed a lumped-element model of the TW displacement during passive state through its coupling to the ATM and PTM (Figure 1C). The displacement was considered in the time domain. The parameters *M*_−_, *c*_−_ and *k*_−_ denoted on the model are the effective mass, stiffness and damping parameters, respectively, whose subscripts ATM, PTM and TW show which part of the tree cricket ear they belong to. The angle *θ* is for the natural slant of the tympana, and has a magnitude of *θ* = 60°.^10^ The system is excited by the sound stimuli (see Figure 1C).

$$F_{1}\left(t\right)=\left(P_{0}\times A_{\mathrm{ATM}}\right)\sin\left(\omega t\right),$$

and

$$F_{2}\left(t\right)=\left(P_{0}\times A_{\mathrm{PTM}}\right)\sin\left(\omega t+\varphi \right)$$

acting on ATM and PTM, respectively, where *P*_0_ = 40 mPa is the sound stimulus amplitude, *A*_−_ is the cross-sectional area of the membrane, *ω* = 2*πf* is the angular frequency with *f* = 3 kHz, and *φ* is the phase angle.

The mathematical equivalent of the system is given in supplemental information, Methods S2.

The ATM parameter values were taken the same as above. Similar to the ATM, the PTM was also assumed to have an elliptic shape, with its precise maximum width and length of 3.5 × 10^−4^ and 8 × 10^−4^, respectively.^10^ The mass of PTM was calculated as for ATM. Since the LDV recordings of the PTM sound response given by Mhatre et al.^10^ demonstrated a flat response with a sensitivity equal to ATM, we took the effective stiffness *k*_*PTM*_ to be the same as *k*_*ATM*_. Likewise, *c*_*ATM*_ and *c*_*PTM*_ were obtained as in the Section above at the considered frequency *f* = 3 kHz. The precise parameter values used for the ATM and PTM are listed in Table 2.

Although the empirical measures of the *O. henryi* material parameters are not available, detailed anatomical measurements of the related field cricket *Gryllus bimaculatus* showed a comparable thickness between the PTM and the TW.^17^ Based on this assumption, we took the *O. henryi* TW thickness to be the same as the thickness of its PTM, namely 2 μm. The length of the *O. henryi* TW was measured as 130 μm (Figure 1G in^10^). As we are interested in the region of TW connected to the tympana, the width was taken as the width of the ATM, namely 900 μm. Hence, assuming a rectangular cross-sectional area for TW and using Equation 7, the effective mass of the TW was calculated as

$$M_{TW}=2.808\times 10^{-10}\;\text{kg.}$$

The effective damping parameter was taken to be the same as for ATM and PTM in both *x* − and *z* − directions (Figures 1B and 1C). For the remaining unknown parameter, the effective stiffness of the TW, we used parameter sweeps by comparing the numerical TW sensitivity with the OCT recordings of the tree cricket species *Oecanthus californicus*, whose passive state tympana also gave a flat response to sound within the measured frequency range 3–4.5 kHz, with a comparable sensitivity to *O. henry*^16^ (see supplemental information, Methods S3). The parameter values adopted for TW are listed in Table 2.

### Quantification and statistical analysis

There are no quantification or statistical analyses to include in this study.

## Supplemental information

Document S1. Figures S1–S8, Tables S1–S5, and Methods S1–S7

Data S1. An animation of the elliptic trace with respect to the phase difference between the tympana of the same ear
