# Supplementary material for: A theoretical description of the tree cricket ear as a highly phase sensitive biomechanical interferometer
Source: iScience. 2026 Sep 4;29(9):117431. doi: 10.1016/j.isci.2026.117431 (PMC13571973; doi:10.1016/j.isci.2026.117431)
Supplement: Supplementary file 1 [file mmc1.pdf]

iScience, Volume *29*

Supplemental information

A theoretical description of the tree cricket ear as a highly phase sensitive biomechanical interferometer

Emine Celiker, Gregory Patrick Sutton, and Natasha Mhatre

Methods S1 Tympana Material Parameters

Methods S1.1 Tympanum effective stiffness validation

As a validation for the effective stiffness value calculated for the anterior tympanic membrane (ATM), we considered the static stiffness of an idealised tympanum - a clamped circular plate. The static stiffness was calculated using the formula

$$k_s = \frac{F}{\delta},$$

where F =is the total force applied to the plate , and

$$\delta = \frac{Pr^4}{64D}$$

is the maximum deflection of the plate, with P=pressure per unit area, r =plate radius and D =bending stiffness ([4], Table 11.2, Condition 10b). The bending stiffness is calculated as $D = \frac{Eh^3}{12(1-\nu^2)}$, where E, h and ν are the Elastic modulus, thickness and Poisson's ratio, respectively, as listed in Table S1. The thickness is the same as the tree cricket ATM thickness [1]. Since the tree-cricket tympana are elliptic, the plate radius was taken as the averaged major and minor axes of the measured dimensions [1]. The radius was then multiplied by 0.27 to reflect that only a small part of the plate is active [2]. Poisson's ratio was taken as a value generally used for insect tympana [3]. The Elastic modulus of tree cricket tympanum is not known, hence we carried out parameter sweeps of the static plate stiffness within known bounds of insect cuticle, [7], until there was a good match with the effective stiffness used for the lumped element model.

Table S1: Parameter values used to calculate the static stiffness of an idealised tympanum.

Parameter	Value
Thickness (h)	2 μm
Radius (r)	84.375 μm
Poisson's Ratio (ν)	0.3
Elastic Modulus (E)	2 GPa

Substituting the values given in Table S1 into the formula, and noting that total force $F = P\pi r^2$ [5], we obtained $k_S = 41.38$ N/m. Hence, the stiffness of the idealised tympanum compared remarkably well with the effective stiffness of the tree cricket ATM ($k = 41.23$ N/m).

Methods S1.2 Tympanum damping

To calculate the frequency dependent damping of the tree cricket ATM, the analytical solution of the equation of motion was utilised. For this, we considered equation (1) which represents the displacement magnitude as obtained from the analytical solution

$$|X(\omega)| = \frac{P_0 \times A}{\omega\sqrt{c^2 + (\omega M - k/\omega)^2}}, \tag{1}$$

where M, k and c are the mass, stiffness and damping parameters of the tympanum; the dependent variable $X(\omega)$ is the complex displacement with respect to the angular frequency ($w = 2\pi f$, f =frequency), $P_0 = 0.04$ Pa and A is the cross-sectional area of the membrane. To match the experimental data, f was considered in the range 2-7 kHz, with a resolution of 0.0625 kHz.

Rearranging equation (1), we obtain the damping as

$$c(\omega) = \sqrt{\left(\frac{A}{\omega}\frac{P_0}{|X(\omega)|}\right)^2 - (\omega M - k/\omega)^2}. \quad (2)$$

For consistency with the effective stiffness calculation, a tympanal sensitivity of $\frac{|X(\omega)|}{P_0} = 6$ nm/Pa was adopted from the experimental LDV recordings [6]. Substituting this into equation (2) gives

$$c(\omega) = \sqrt{\left(\frac{A}{(6\times 10^{-9})\omega}\right)^2 - (\omega M - k/\omega)^2}. \quad (3)$$

Equation (3) was used as a starting point for the effective damping parameter sweeps. For this, the constant multiples of equation (3) were applied to calculate the ATM displacement magnitude and phase while keeping the effective mass and stiffness fixed. The obtained results were first compared with the LDV data [6] schematically.

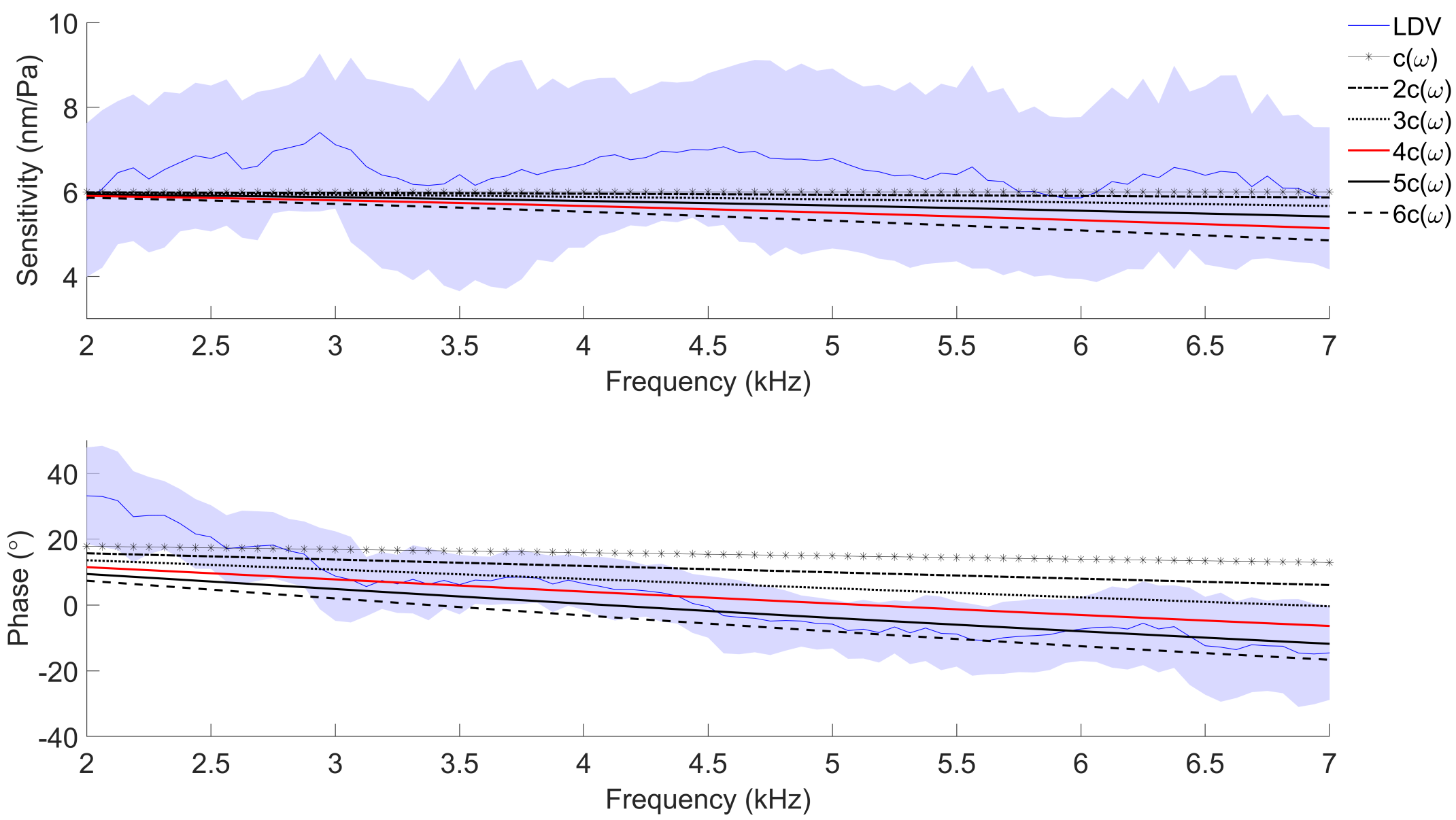

Figure S1: A comparison of the numerical and experimental ATM displacement magnitude (top figure) and phase (bottom figure), for constant multiples of the damping parameter $c(\omega)$. The LDV data are represented as mean +/- SD.

As demonstrated from Figure S1, the numerical sensitivity calculations remained within the standard deviation bounds of the LDV recordings. To obtain the best fit, the percentage errors were calculated using the relative error formula

$$RE = \frac{1}{N}\sum_{i=1}^{N}\frac{|y_i - \hat{y}_i|}{|y_i|}100,$$

where $y_i, \hat{y}_i$, $i = 1, 2, \cdots N$ are the mean experimental and numerical data, respectively, and N is the number of data points. The obtained results are presented in Table S2.

Table S2: Relative percentage error (RE) between the mean LDV recordings [6] and numerical results for sensitivity comparison.

	RE (%)
$c(\omega)$	7.7
$2c(\omega)$	8.4
$3c(\omega)$	9.7
$4c(\omega)$	11.5
$5c(\omega)$	13.5

For a comparison between the experimental and numerical phase data, despite a calibration of 20° of the numerical data to account for differences with the experimental set-up, the results fell out of the standard deviation bounds for all considered damping parameters (Figure S1). Nevertheless, for $4c(\omega)$, the numerical phase data was within bounds for 2.5 - 7 kHz for the 2- 7 kHz frequency range considered. Since the numerical phase data had regions out of the standard deviation bounds, rather than calculating the percentage error, we calculated the maximum absolute difference between the two data sets. The obtained results did not show a significant difference between the considered damping parameter values (Table S3).

Table S3: Maximum absolute difference between the phase data of the mean LDV recordings (E_p) [6], and the numerical results (N_p).

	max $\lvert E_p - N_p \rvert$ (°)
$c(\omega)$	27.8
$2c(\omega)$	24.2
$3c(\omega)$	26.1
$4c(\omega)$	28.0
$5c(\omega)$	29.7

Based on the schematic investigation (Figure S1) and the following percentage error calculation for sensitivity (Table S2) and maximum absolute difference for the phase data (Table S3), the frequency dependent parameter values were adopted as $4c(\omega)$. The range of values of $4c(\omega)$ is given in Figure S2.

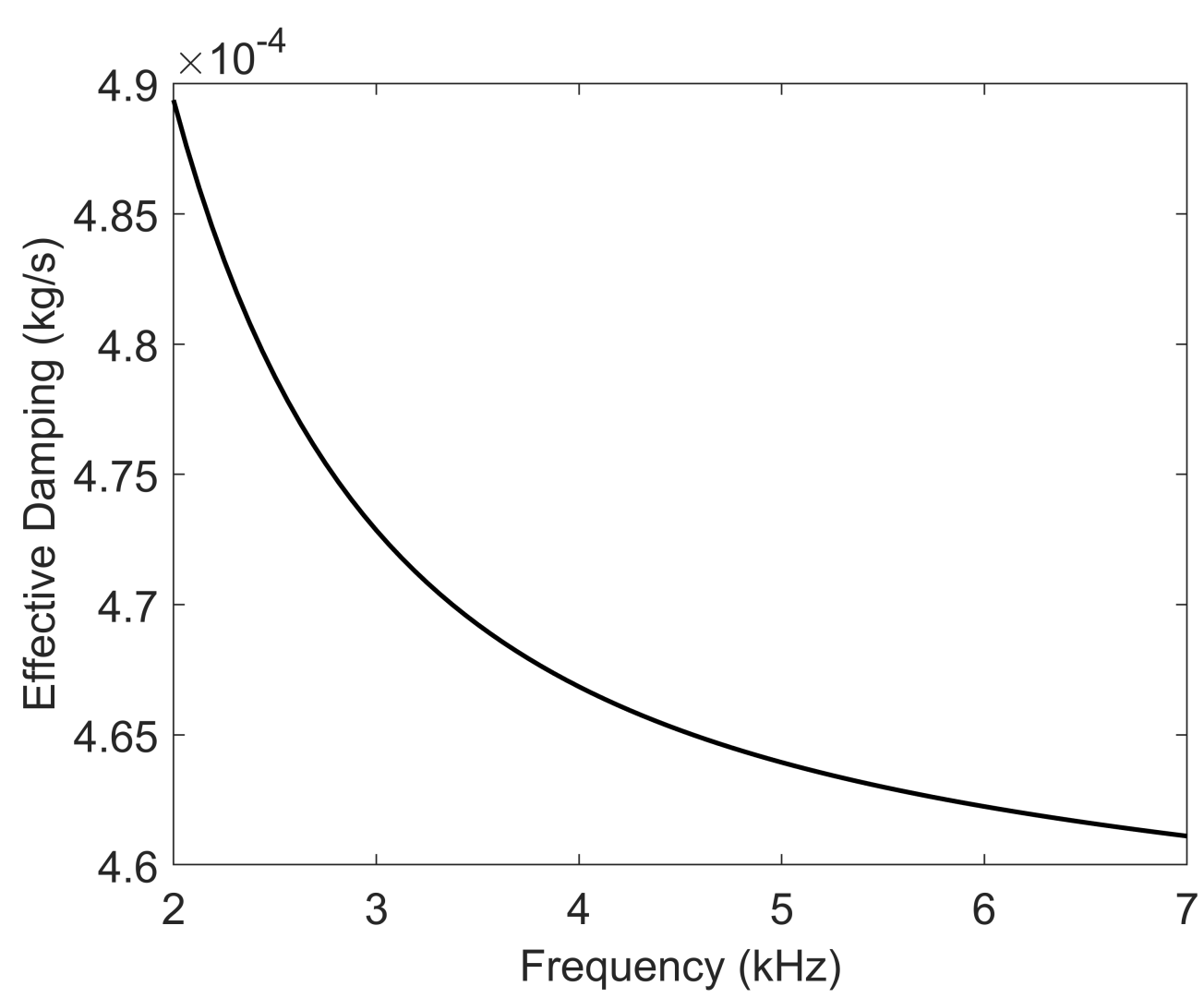

Figure S2: The frequency dependent effective damping values.

Methods S2 Mathematical representation of tree cricket ear biomechanism

The mechanical system in Figure 2b (Main text, Section 2.2) is mathematically described by the following system of second-order ordinary differential equations.

$$
\begin{aligned}
M_{ATM}x_1''(t) = F_1(t) \quad &+ \quad k_{ATM}\left(\sqrt{(x_3(t)-x_1(t))^2+z(t)^2}-\sqrt{(x_3(0)-x_1(0))^2+(z(0))^2}\right)\cos(\theta)\\
&+ \quad c_{ATM}\frac{d}{dt}\left(\sqrt{(x_3(t)-x_1(t))^2+z(t)^2}\right)\cos(\theta)+k_{ATM}(x_1(0)-x_1(t))-c_{ATM}x_1'(t)\\
M_{PTM}x_2''(t) = -F_2(t) \quad &- \quad k_{PTM}\left(\sqrt{(x_2(t)-x_3(t))^2+z(t)^2}-\sqrt{(x_2(0)-x_3(0))^2+(z(0))^2}\right)\cos(\theta)\\
&- \quad c_{PTM}\frac{d}{dt}\left(\sqrt{(x_2(t)-x_3(t))^2+z(t)^2}\right)\cos(\theta)+k_{PTM}(x_2(0)-x_2(t))-c_{PTM}x_2'(t)\\
M_{TW}x_3''(t) = \quad &- \quad k_{ATM}\left(\sqrt{(x_3(t)-x_1(t))^2+z(t)^2}-\sqrt{(x_3(0)-x_1(0))^2+(z(0))^2}\right)\cos(\theta)\\
&- \quad c_{ATM}\frac{d}{dt}\left(\sqrt{(x_3(t)-x_1(t))^2+z(t)^2}\right)\cos(\theta)\\
&+ \quad k_{PTM}\left(\sqrt{(x_2(t)-x_3(t))^2+z(t)^2}-\sqrt{(x_2(0)-x_3(0))^2+(z(0))^2}\right)\cos(\theta)\\
&+ \quad c_{PTM}\frac{d}{dt}\left(\sqrt{(x_2(t)-x_3(t))^2+z(t)^2}\right)\cos(\theta)-k_{TW}x_3(t)-c_{TW}x_3'(t)\\
M_{TW}z_3''(t) = \quad &- \quad k_{ATM}\left(\sqrt{(x_3(t)-x_1(t))^2+z(t)^2}-\sqrt{(x_3(0)-x_1(0))^2+(z(0))^2}\right)\sin(\theta)\\
&- \quad c_{ATM}\frac{d}{dt}\left(\sqrt{(x_3(t)-x_1(t))^2+z(t)^2}\right)\sin(\theta)\\
&- \quad k_{PTM}\left(\sqrt{(x_2(t)-x_3(t))^2+z(t)^2}-\sqrt{(x_2(0)-x_3(0))^2+(z(0))^2}\right)\sin(\theta)\\
&- \quad c_{PTM}\frac{d}{dt}\left(\sqrt{(x_2(t)-x_3(t))^2+z(t)^2}\right)\sin(\theta)\\
&- \quad k_{TW}z(t)-c_{TW}z'(t),
\end{aligned}
$$

with initial conditions

$$x_1(0) = 0 \text{ m}; x_2(0) = 0.00022 \text{ m}; x_3(0) = 0.00013 \text{ m}; z(0) = 0.000085 \text{ m}$$

and

$$x_1'(0) = x_2'(0) = x_3'(0) = z'(0) = 0.$$

The initial conditions were based on the precise ear measurements given in [1].

The system of equations was solved numerically using the function "ParametricNDSolve" in Wolfram Mathematica 13.3 [9].

Methods S3 Trachea wall effective stiffness

Since the stiffness of the tree cricket trachea wall has not been empirically quantified, we used numerical parameter sweeps to determine a realistic effective stiffness (k_{TW}) value at 3 kHz. As a simplifying assumption, we took k_{TW} to be the same in the $x-$ and $z-$ directions. For the starting point of the parameter sweeps, we used the effective stiffness of the ATM $k = 41.23$ N/m. The parameter sweeps considered the constant multiples of k. The constant multiples were substituted to the mathematical system outlined in Section Methods S2, while keeping the remaining parameters fixed. The simulations were considered at 3 kHz, and the phase difference between the tympana was varied within the range $0° < \varphi < 180°$ for the maximum displacement magnitude of the wall.

As a benchmark for the parameter sweeps, the numerical results were compared to the Optical Coherence Tomography (OCT) recordings of the trachea wall displacement magnitude in the passive state tree cricket ear, given by Mhatre et al. [8]. The OCT recordings showed a sensitivity of $\sim$4nm/ Pa in the $x-$direction and $\sim$3nm/ Pa in the $z-$directions.

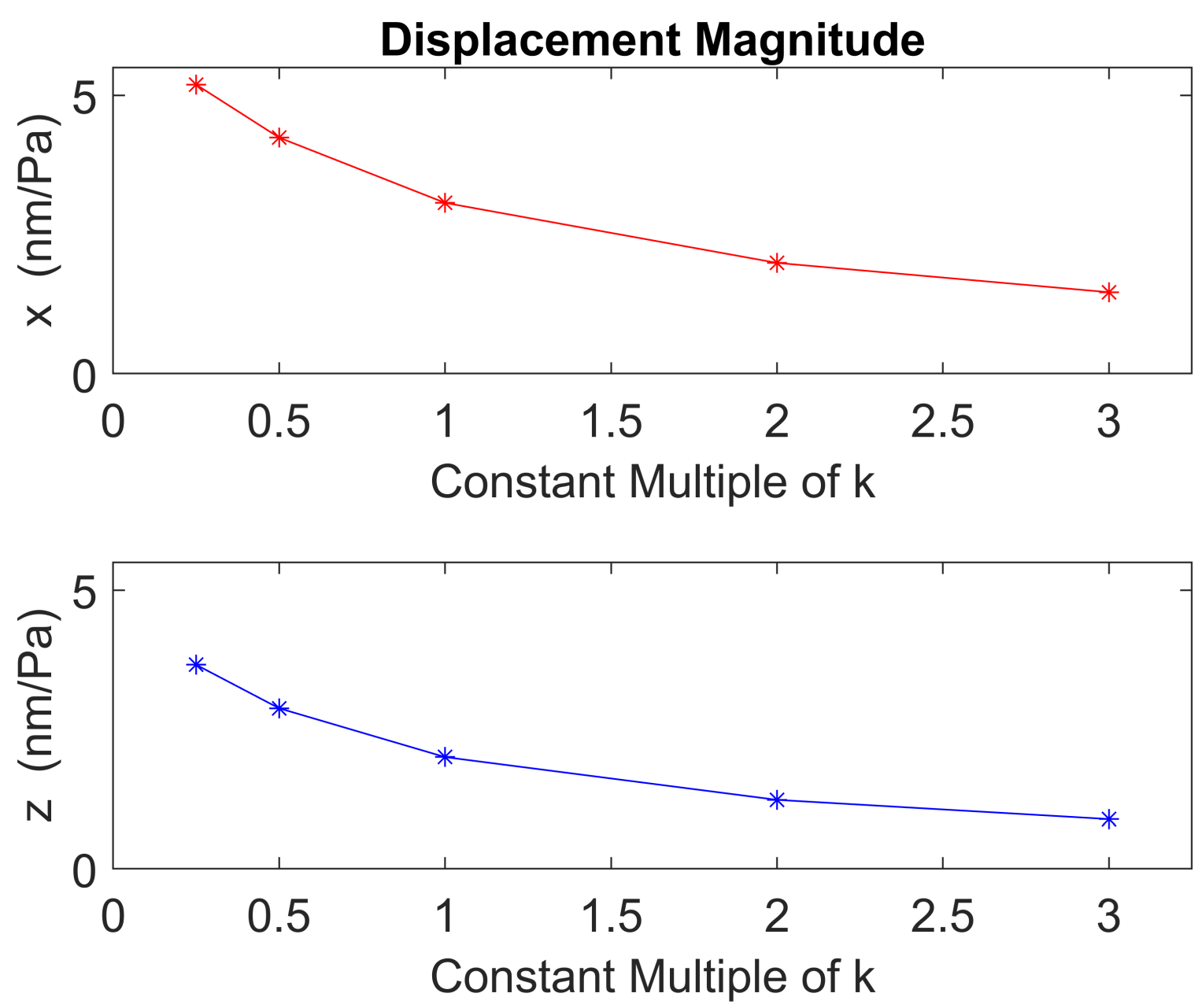

Figure S3: The numerical maximal displacement magnitude of the TW in the $x-$direction (top figure) and $z-$direction (bottom figure), for constant multiples of the effective stiffness k.

The numerical results for various constant multiples of $k = 41.23$ N/m are given Figure S3. Following a comparison with the experimental data, the effective stiffness of the trachea wall was adopted as $k_{TW} = \frac{1}{2}k = 20.62$ N/m.

Methods S4 Trachea wall displacement with respect to sound pressure magnitude differences at the tympana

To investigate the effect of sound magnitude differences at ATM and PTM on the TW displacement, we simulated the wall movement in the time domain by first assuming an equal force of 0.04 Pa acting on the tympana, followed by a difference of 0.01 Pa between the magnitudes (assuming a sound pressure of 0.05 Pa on the ATM and 0.04 Pa on the PTM). The results demonstrated that when the sound arriving to the tympana are *in-phase* ($\varphi = 0°$) or *out-of-phase* ($\varphi = 180°$), the TW did not have an elliptic displacement (Figure S4).

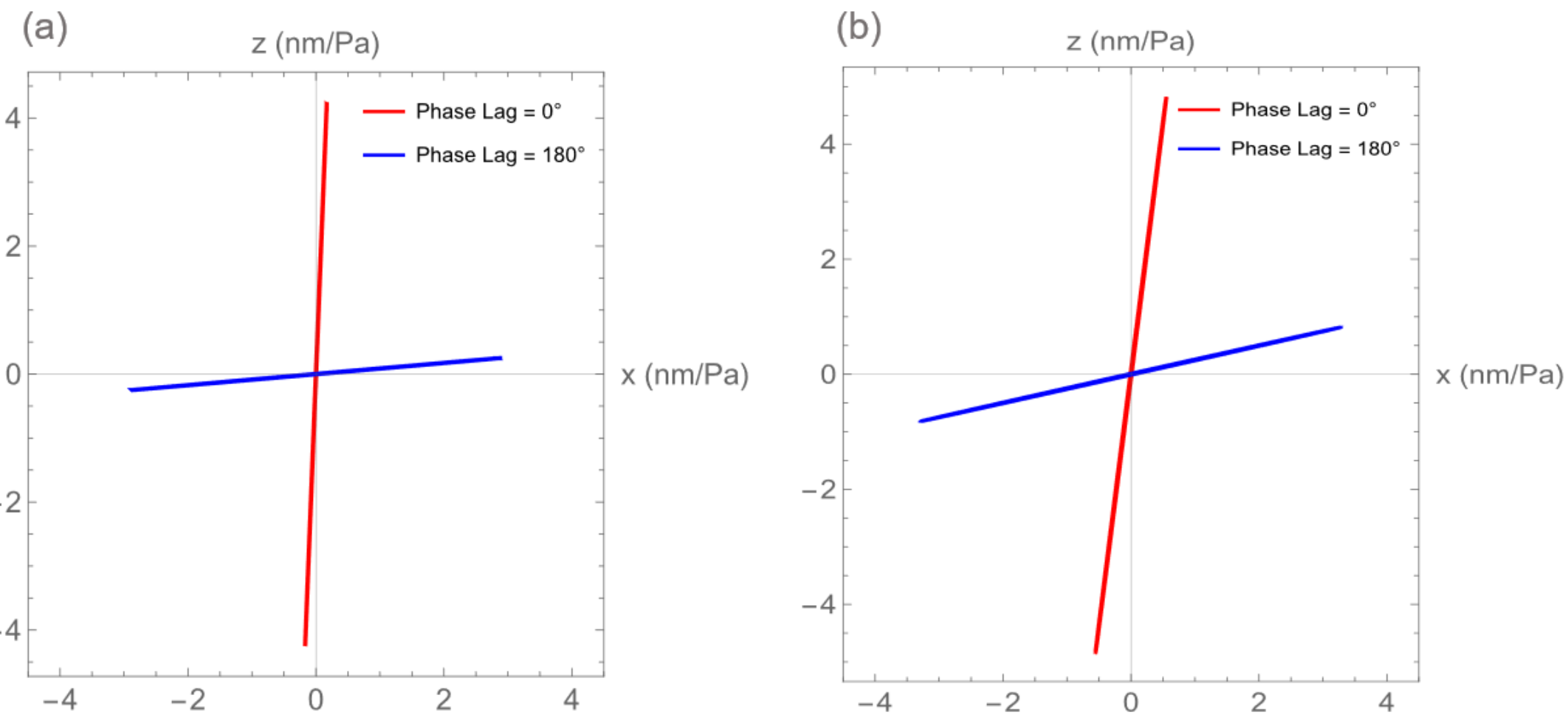

Figure S4: The displacement pattern of the TW when the ATM and PTM are acted on by forces *in-phase* (phase lag= 0°) and *out-of-phase* (phase lag= 180°) of (a) equal magnitude (0.04 Pa), and (b) unequal magnitude.

To test how unequal sound pressure magnitudes with phase lags ($\varphi = 45^o, 90^o, 135^o$) affect TW displacement, we evaluated three cases with varying pressure magnitudes between the ATM and PTM. The obtained results showed that when the pressure magnitudes are comparable, the TW exhibits distinct elliptic displacements (Figure S5a). As the magnitude at the PTM decreases relative to the ATM, these ellipses begin to tilt and narrow (Figure S5b). Finally, when the pressure magnitude at the PTM becomes negligible, the ellipses were significantly narrowed and aligned, eliminating the phase-lag dependence observed in the previous cases (Figure S5c).

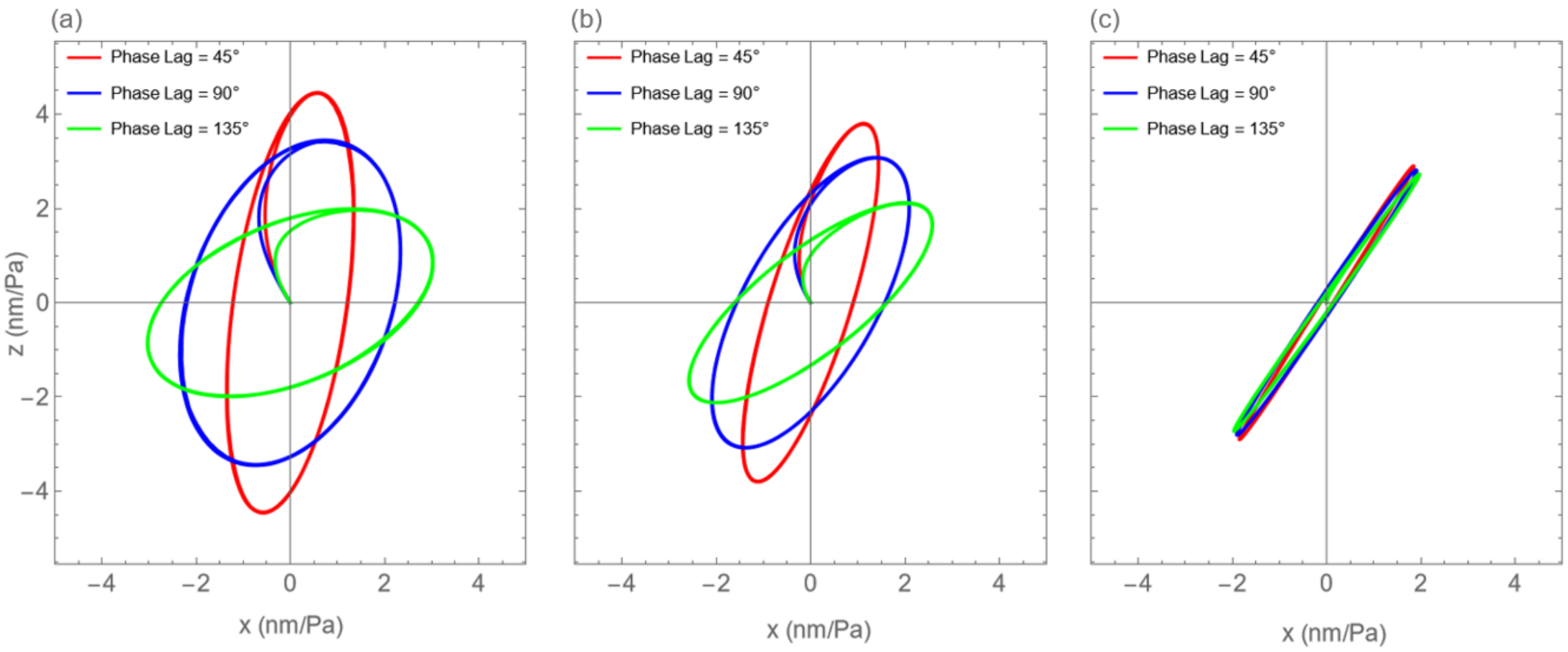

Figure S5: The displacement pattern of the TW when the tympana are acted on by forces with phase lags and unequal magnitudes of (a) 0.05 Pa (ATM) and 0.04 Pa (PTM), (b) 0.05 Pa (ATM) and 0.025 Pa (PTM), and (c) 0.05 Pa (ATM) and 0.0025 Pa (PTM).

Methods S5 Tympanum dynamic response analysis

In the time domain, we simulated the ATM and PTM response to sound pressure at 3 kHz and a phase lag of $\varphi = \pi/2$ between the tympana, using the fully-coupled system outlined in Section Methods S2. The results showed a flat response from both ATM and PTM, with small differences between amplitudes: ATM Amplitude = 5.13 nm/Pa, PTM Amplitude = 4.56 nm/Pa (Figure S6).

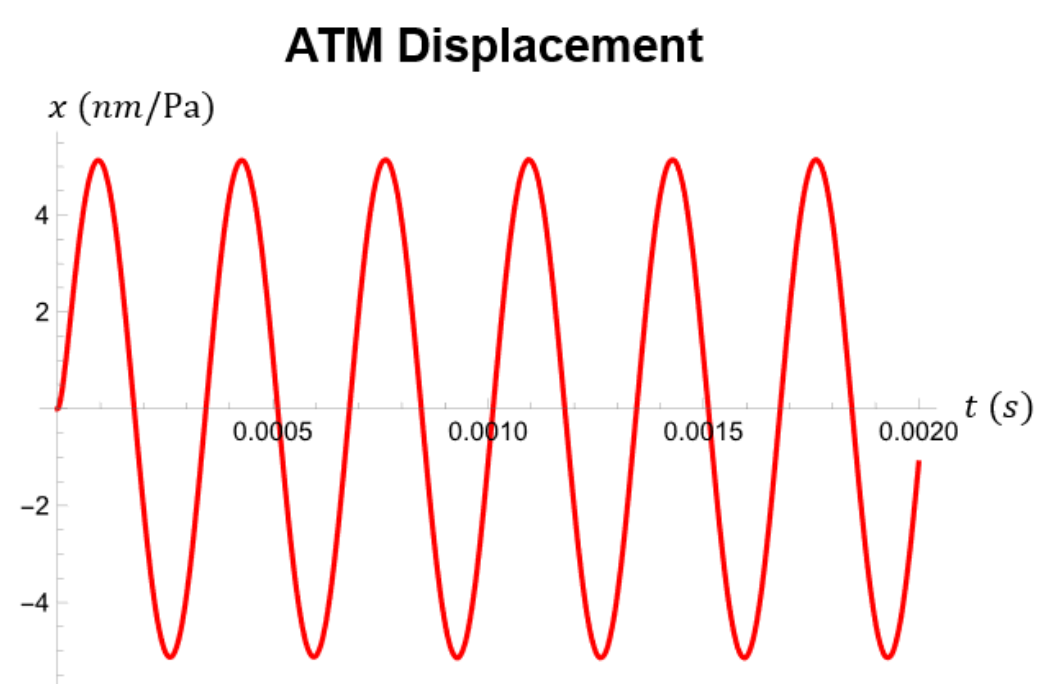

(a) ATM response to sound pressure in the time domain.

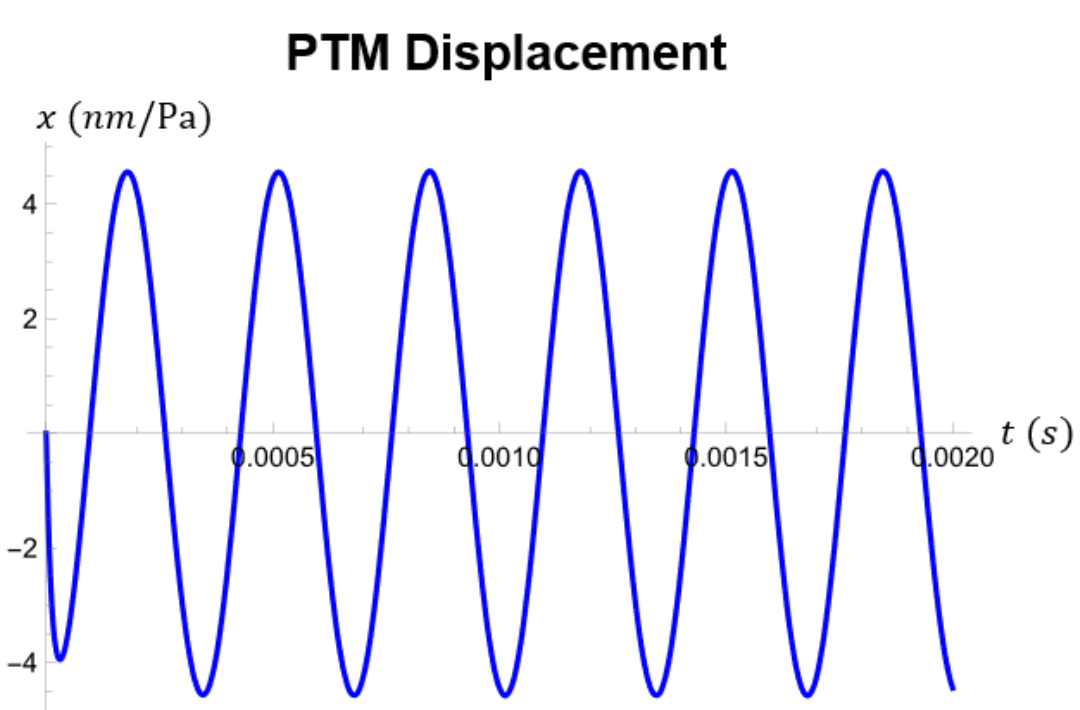

(b) PTM response to sound pressure in the time domain.

Figure S6: The displacement of ATM and PTM obtained using a fully-coupled model.

Methods S6 Parametric sensitivity analysis of the coupled lumped-element model

To test the sensitivity of the developed model to the effective stiffness, mass and damping parameters, we carried out parameter sweeps by systematically varying their values within a range of ±25% (Figure S7).

Varying the TW damping (c_{TW}) by up to ±25%, while keeping the other parameters fixed did not lead to a significant change in the TW displacement magnitude (Figure S7a). A similar result was obtained when the TW mass (M_{TW}) was varied while keeping the other parameters fixed (Figure S7b). These results suggest that the system's response is insensitive to the damping and mass of the TW within the tested range.

In contrast, an analysis of the TW stiffness (k_{TW}) showed that a decrease in stiffness resulted in larger displacement magnitudes of the TW in both the $x-$ and $z-$ directions (Figure S7c, top), while an increase led to smaller magnitudes (Figure S7c, bottom). Nevertheless, the TW displacement retained its qualitative behaviour, namely the increase of the displacement magnitude in the $x-$axis with the increase of the phase lag between the ATM and PTM, and the reverse relationship in the $z-$axis. This strongly suggests that, although the system is stiffness dominated, it is robust to parameter variations.

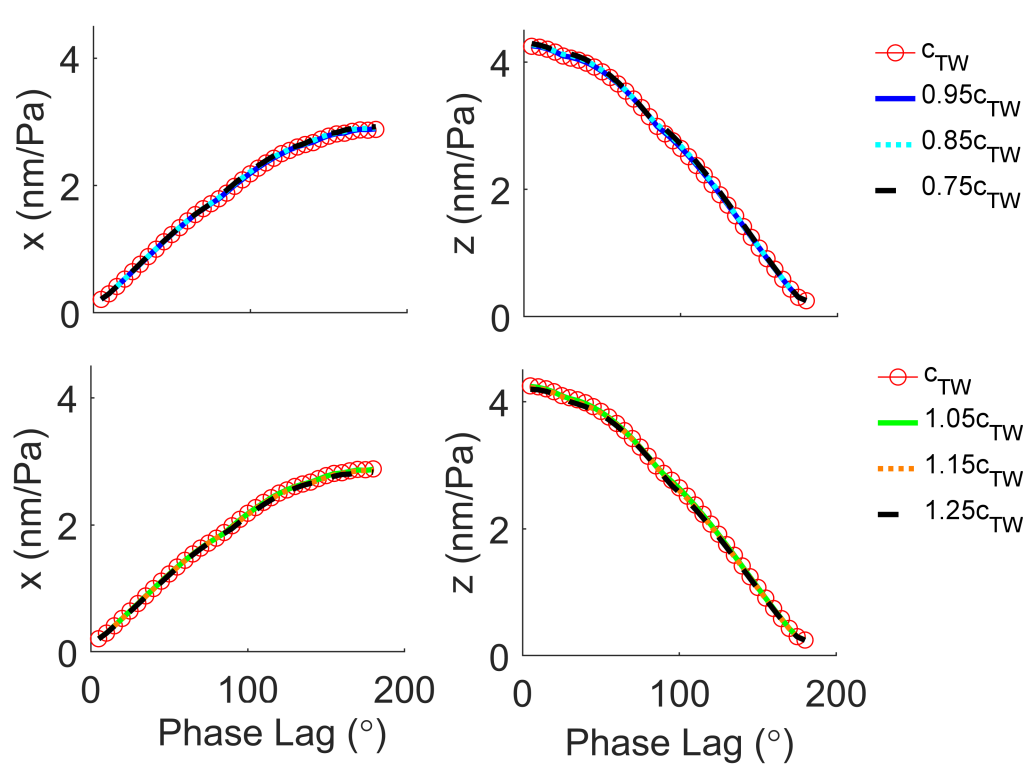

(a) TW effective damping (c_{TW}) variation.

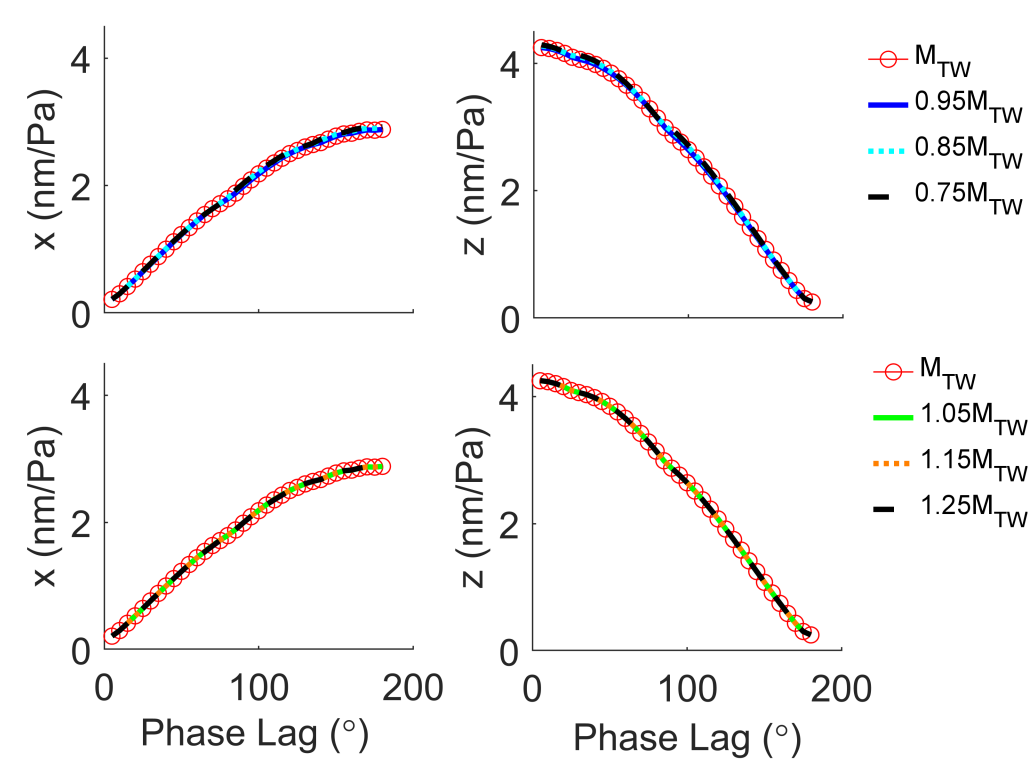

(b) TW effective mass (M_{TW}) variation.

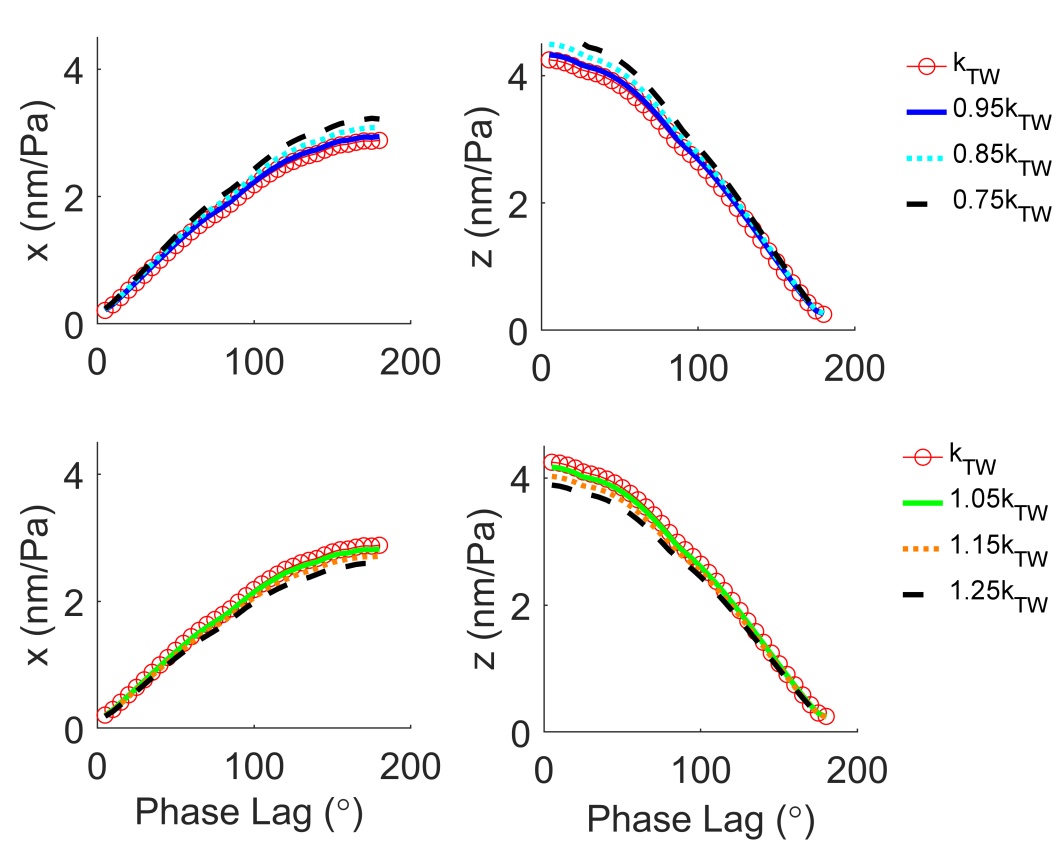

(c) TW effective stiffness (k_{TW}) variation.

Figure S7: The displacement of the trachea wall (TW) via the lumped-element model based on differing effective (a) damping, (b) mass, and (c) stiffness values.

Methods S7 Finite Element Analysis of Phase Difference Between the Anterior and Posterior Tympana

To numerically investigate the phase difference magnitude between the ATM and PTM of the same ear, we carried out finite element simulations of the sound arriving to the ipsilateral and contralateral ears. An idealized geometry of a tree cricket resting on a substrate was placed in a spherical domain representing a truncated free-field of air (Figure S8A,B). The sphere had a radius of 45 mm, with a layer of 10 mm thickness from its boundary assigned a Perfectly Matched Layer (PML) condition to absorb any reflections from the truncation of the computational grid.

Within the sphere, the solution to the Helmholtz equation was considered in the frequency domain, by assigning the *Pressure Acoustics, Frequency Domain* node of COMSOL Multiphysics, v. 6.1 [10]. The sound stimulus had a magnitude of 0.02 Pa, or 60 dB SPL re 2×10^{-5} N/m^2, and was set-up as background pressure. The incidence angles considered were $0^o, 30^o, 60^o$, and 90°, where 0° is the front of the insect, moving in a counter-clock wise direction (Figure S8A). The substrate boundary was assigned an impedance boundary condition, with a specified variation of absorption coefficients (0.01-0.4) based on empirical investigations in the literature for tree bark and plants ([11]-[13]). Although the absorption coefficient of insect cuticle is unknown, we approximate its effect by investigating a range of absorption coefficients known to occur in natural materials. The surface of the idealized tree cricket geometry was assigned the same absorption coefficient as the substrate for simplicity. Both the substrate and the tree cricket surface were also assigned the thermoviscous acoustics boundary condition to account for the potential energy loss from thermal and viscous boundary layers forming naturally around these surfaces. The solution was considered for the frequency range 1.5 - 7 kHz, with a resolution of 1.5 kHz.

The solution to the mathematical system described above was obtained using the finite element method and the Simulation Toolbox COMSOL Multiphysics v. 6.1 [10]. The surface of the idealized insect and the substrate employed a mesh size with a largest diameter of 9×10^{-4} mm. A tetrahedral mesh was constructed within the whole sphere with a maximum element diameter of 7.2 mm, and connected conformally with the boundary mesh. This mesh allowed for at least six elements per wavelength at the largest considered frequency of 7 kHz.

To calculate the phase angle, the complex sound pressure were recorded at one point on each of the tympana (Figure S8B). All the points of measurement were parallel to the y-axis, and on the plane $x = 5$, parallel to the yz-plane (Figure S8C). The phase angle was then obtained using the well-known transformation

$$\varphi = \arctan(p_{im}/p_{re}),$$

based on the complex sound pressure $p = p_{re} + ip_{im}$, where $i = \sqrt{-1}$. Finally the phase difference was obtained by subtracting the calculated phase angle at the ATM and PTM of the same leg.

Our parameter sweep of the absorption coefficient at 3 kHz and 90° incidence angle demonstrated a significant phase difference between the ATM and PTM even with the smallest absorption (Table S4 and Figure S8F). Further analysis of the results with a fixed absorption coefficient of 0.2 demonstrated the large influence of the insect's body on the phase angle (Figure S8D). Moreover, the frequency sweep with a constant absorption coefficient of 0.2 demonstrated a decrease in the phase difference between the ipsilateral ATM and PTM. The numerical results also showed an increase in the phase difference between the tympana of the ipsilateral ear with the increase of the sound incidence angle (Table S5).

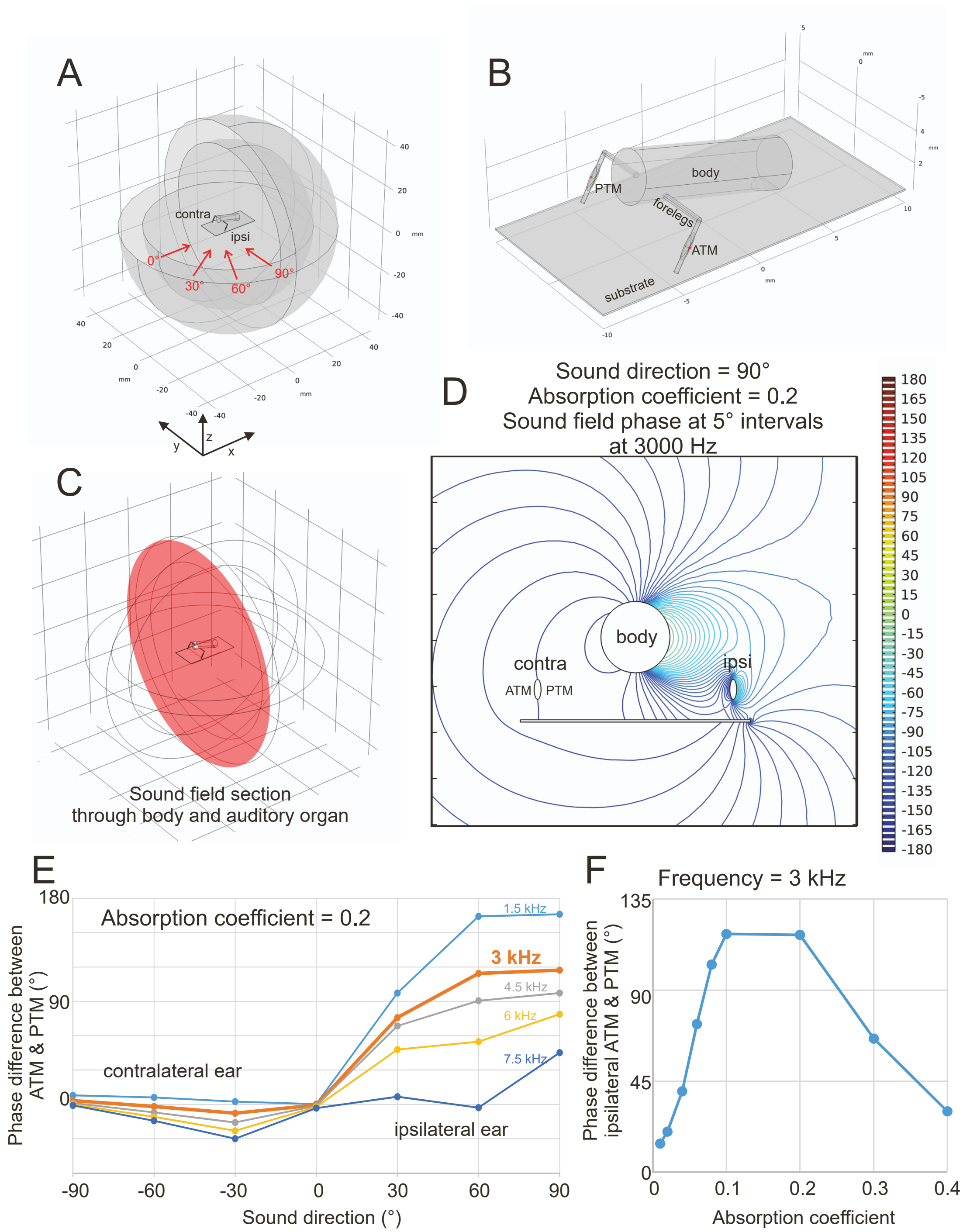

Figure S8: **Finite element analysis (FEA) of diffraction and absorption effects from an idealized tree-cricket body.** A. The computational domain and the sound incidence angles. B. The idealized tree cricket body on a substrate. C. The plane $x = 5$ on which the phase calculations were carried out. D. A contour plot of the phase on the plane defined in C. E. The change in the phase difference between the tympana with respect to incidence angles and frequency. The change in the phase difference between the tympana at 3 kHz with respect to the absorption coefficient.

Table S4: Phase difference variations between the tympana of the ipsilateral ear (IE), contralateral ear (CE), and between the ears, with respect to the absorption coefficient.

Abs. Coeff.	ATM - PTM IE (°)	ATM - PTM CE (°)	Diff. Between Ears (°)
0.01	14.24	13.92	0.32
0.02	20.07	12.14	7.93
0.04	93.99	9.64	30.35
0.06	73.24	7.96	65.28
0.08	102.65	6.76	95.89
0.1	117.73	5.85	111.88
0.2	117.29	3.33	113.95
0.3	66.10	2.15	63.94
0.4	30.16	1.45	28.71

Table S5: Phase difference variations between the tympana of the ipsilateral ear (IE) and the contralateral ear (CE) with respect to the incidence angles for an absorption coefficient of 0.2.

Angle of Incidence (°)	ATM - PTM IE (°)	ATM - PTM CE (°)
0	-0.544	-0.561
30	75.831	-7.689
60	114.344	-1.799
90	117.287	3.335

References

[1] Mhatre, N., Montealegre-z, F., Balakrishnan, R., & Robert, D. (2009). Mechanical response of the tympanal membranes of the tree cricket *Oecanthus henryi*. *Journal of Comparative Physiology A*, **195**, 453-462.

[2] Hauer, B. D., Doolin, C., Beach, K. S. D., & Davis, J. P. (2013). A general procedure for thermomechanical calibration of nano/micro-mechanical resonators. *Annals of Physics*, **339**, 181-207.

[3] Malkin, R., McDonagh, T. R., Mhatre, N., Scott, T. S., & Robert, D. (2014). Energy localization and frequency analysis in the locust ear. *Journal of The Royal Society Interface*, **11**(90), 20130857.

[4] Young, W. C., Budynas, R. G., & Sadegh, A. M. (2002). *Roark's formulas for stress and strain* (Vol. 7). New York: McGraw-hill.

[5] Michelsen, A., & Larsen, O. N. (1978). Biophysics of the ensiferan ear: I. Tympanal vibrations in bushcrickets (Tettigoniidae) studied with laser vibrometry. *Journal of comparative physiology*, **123**(3), 193-203.

[6] Mhatre, N., & Robert, D. (2013). A tympanal insect ear exploits a critical oscillator for active amplification and tuning. *Current Biology*, **23**(19), 1952-1957.

[7] Vincent, J. F., & Wegst, U. G. (2004). Design and mechanical properties of insect cuticle. *Arthropod structure & development*, **33**(3), 187-199.

[8] Mhatre, N., Dewey, J. B., Quiñones, P. M., Mason, A., Applegate, B. E., & Oghalai, J. S. (2021). Reconstruction of sound driven, actively amplified and spontaneous motions within the tree cricket auditory organ. *bioRxiv, 2021-11.*

[9] Wolfram Research, Inc., Mathematica, Version 13.3, Champaign, IL (2023).

[10] COMSOL Multiphysics® v. 6.1. www.comsol.com. COMSOL AB, Stockholm, Sweden.

[11] D'Alessandro, F., Asdrubali, F., & Mencarelli, N. (2015). Experimental evaluation and modelling of the sound absorption properties of plants for indoor acoustic applications. *Building and environment*, **94**, 913-923.

[12] Li, M., Van Renterghem, T., Kang, J., Verheyen, K., & Botteldooren, D. (2020). Sound absorption by tree bark. *Applied acoustics*, **165**, 107328.

[13] Jang, E. S. (2023). Sound absorbing properties of selected green material—A review. *Forests*, **14**(7), 1366.
